# Integrating CRISPR-Enabled Trackable Genome Engineering and Transcriptomic Analysis of Global Regulators for Antibiotic Resistance Selection and Identification in Escherichia coli

**DOI:** 10.1128/mSystems.00232-20

**Published:** 2020-04-21

**Authors:** Cong Chen, Alaksh Choudhury, Shuanghong Zhang, Andrew D. Garst, Xin Song, Xunli Liu, Tao Chen, Ryan T. Gill, Zhiwen Wang

**Affiliations:** a Key Laboratory of Systems Bioengineering (Ministry of Education), SynBio Research Platform, Collaborative Innovation Center of Chemical Science and Engineering (Tianjin), School of Chemical Engineering and Technology, Tianjin University, Tianjin, China; b Department of Chemical and Biological Engineering, University of Colorado Boulder, Boulder, Colorado, USA; c College of Forestry, Shandong Agricultural University, Taian, Shandong Province, China; d Inscripta Inc., Boulder, Colorado, USA; University of Illinois at Chicago

**Keywords:** antibiotic resistance, global regulators, CREATE, transcriptome, mutation

## Abstract

It is important to expedite our understanding of antibiotic resistance to address the increasing numbers of fatalities and environmental pollution due to the emergence of antibiotic resistance and multidrug-resistant strains. Here, we combined the CRISPR-enabled trackable genome engineering (CREATE) technology and transcriptomic analysis to investigate antibiotic tolerance in Escherichia coli. We developed rationally designed site saturation mutagenesis libraries targeting 23 global regulators to identify fitness-conferring mutations in response to diverse antibiotic stresses. We identified seven novel mutations that confer resistance to the ribosome-targeting antibiotics doxycycline, thiamphenicol, and gentamicin in E. coli. To the best of our knowledge, these mutations that we identified have not been reported previously during treatment with the indicated antibiotics. Transcriptome sequencing-based transcriptome analysis was further employed to evaluate the genome-wide changes in gene expression in E. coli for SoxR G121P and cAMP receptor protein (CRP) V140W reconstructions, and improved fitness in response to doxycycline and gentamicin was seen. In the case of doxycycline, we speculated that SoxR G121P significantly increased the expression of genes involved in carbohydrate metabolism and energy metabolism to promote cell growth for improved adaptation. In the CRP V140W mutant with improved gentamicin tolerance, the expression of several amino acid biosynthesis genes and fatty acid degradation genes was significantly changed, and these changes probably altered the cellular energy state to improve adaptation. These findings have important significance for understanding such nonspecific mechanisms of antibiotic resistance and developing new antibacterial drugs.

**IMPORTANCE** The growing threat of antimicrobial resistance poses a serious threat to public health care and motivates efforts to understand the means by which resistance acquisition occurs and how this can be combatted. To address these challenges, we expedited the identification of novel mutations that enable complex phenotypic changes that result in improved tolerance to antibiotics by integrating CREATE and transcriptomic analysis of global regulators. The results give us a better understanding of the mechanisms of resistance to tetracycline antibiotics and aminoglycoside antibiotics and also indicate that the method may be used for quickly identifying resistance-related mutations.

## Abstract (precis)

The growing threat of antimicrobial resistance poses a serious threat to public health care and motivates efforts to understand the means by which resistance acquisition occurs and how this can be combatted. To address these challenges, we expedited the identification of novel mutations that enable complex phenotypic changes that result in improved tolerance to antibiotics by integrating CREATE and transcriptomic analysis of global regulators. The results give us a better understanding of the mechanisms of resistance to tetracycline antibiotics and aminoglycoside antibiotics and also indicate that the method may be used for quickly identifying resistance-related mutations.

## INTRODUCTION

The misuse and overuse of multiple antibiotics have caused a rapid increase in the rate of resistance acquisition in clinical and agricultural settings and are presenting unprecedented challenges to the medical industry and animal husbandry and agriculture (1–5). Recent reports from the United Nations indicate that infections caused by antibiotic-resistant bacteria currently claim as many as 700,000 lives globally each year and anticipate that this number will increase dramatically in coming decades (https://news.un.org/en/story/2019/04/1037471). This motivates efforts to understand the underlying causes of this major global concern. While most well-characterized mutations that confer resistance occur directly in the antibiotic binding sites, mutations can also occur in regulatory genes to improve tolerance by changing intracellular antibiotic concentrations by one of two mechanisms (2, 6). In the first mechanism, mutations can downregulate the expression of major porins that facilitate the diffusion of hydrophilic antibiotics. Since the facilitation of transport by porins is nonspecific (7, 8), the downregulation of porin expression in response to one drug may also confer multidrug resistance phenotypes (9–12). Through the second mechanism, the intracellular drug concentrations are reduced by the increased expression of efflux pumps. Mutations often increase the expression of nonspecific efflux pumps with the ability to target a wide range of structurally dissimilar antibiotics, again facilitating multidrug resistance (6). Therefore, studying such nonspecific mechanisms of resistance is extremely important.

Mutations in global regulatory proteins play an important role in the emergence of nonspecific resistance. Winkler et al. recently developed a resistome database with over 5,000 resistance-conferring mutations that improved resistance or tolerance to 200 different stress conditions, including antibiotics (1). The mutations in essential proteins involved in transcription, such as RNA polymerases, the transcription termination protein (Rho), sigma factors, and global regulatory proteins, were significantly enriched across conditions (1, 13). Mutations in global regulators can simultaneously alter the expression levels of many genes to improve adaptation to external environment stress, including antibiotics (14). Antibiotic resistance/tolerance in bacteria is a complex phenotype controlled by multiple genes. Therefore, essential genes involved in global regulation are important targets for the emergence of such complex phenotypes.

Currently, mutations in essential global regulatory proteins are identified mainly by using adaptive laboratory evolution experiments (ALE) and metagenomic sequencing of resistant clinical isolates (13). The identification of these mutations is time- and labor-intensive and often costly. Moreover, the activity of global regulators is extremely complex and spans multiple levels, ranging from the protein level to the regulon level. Therefore, it is not known if mutations within the same gene confer similar fitness effects. The extent of the fitness benefits of mutations across antibiotics is also not known. Finally, global regulators are a part of very complex interaction networks (15), and it is not known if mutations in previously unidentified global regulators could also impact resistance. Answering these questions can significantly improve the design of new antibiotics and the formulation of antibiotic combination therapies.

Recent multiplex genome editing strategies (e.g., multiplexed automated genome engineering [MAGE] [16], TaGTEAM [17, 18], pORTMAGE [19], directed evolution with random genomic mutations [DIvERGE] [20]) provide lucrative platforms for rapid mutagenesis of these genes. One of the most popular approaches for the construction of a genome mutation library in Escherichia coli is bacteriophage lambda Red recombination-based MAGE (16). MAGE improves genome editing with recombineering efficiency and can generate combinatorial mutations at multiple target sites by introducing the same pool of oligonucleotides for recombineering in several repeated cycles (16, 21). However, the host strains need several modifications, such as deletion of methyl-directed mismatch repair (MMR) and DNA primase (dnaG) (16, 22). Recently, DIvERGE has overcome this challenge and does not involve the permanent inactivation of the endogenous mismatch repair system (20). However, with all these approaches, a high degree of library diversity is achieved by repeated transformation cycles in which cells undergo several cycles of heat shock and electroporation. This repeated stress may be detrimental to diversity. Another major challenge with MAGE-like approaches is the lack of trackability. Finally, in order to identify beneficial mutations, one needs to sequence entire genomes after selection, which is costly. Therefore, for thorough investigation, the mutation libraries are often restricted to a few genes.

The newly emerging CRISPR-Cas9 recombineering-mediated high-throughput genome mutagenesis technologies can help address several limitations associated with previous platforms. Garst et al. used the Cas9-mediated recombineering principle to develop a platform for the construction of trackable genome-wide mutation libraries (23). In the CRISPR-enabled trackable genome engineering (CREATE) work flow (see Fig. S1 in the supplemental material), one designs 230-bp editing cassettes that express the guide RNA (gRNA) targeting a genomic locus and that also encode the repair template to provide rescue from Cas9 gRNA-induced cell death. Several such editing cassettes targeting different loci across the genome can be synthesized on DNA microarrays. Subsequent high-throughput cloning and transformation into cells with active Cas9 and bacteriophage lambda Red recombination allow construction of large mutant libraries. Additionally, the 230-bp cassette carried by a plasmid also acts as a barcode to track mutations and assign fitness to different mutations. In a single study, Garst et al. identified 141 new fitness-conferring mutations across diverse conditions (23). The CREATE technology can be used to expedite the identification of fitness-conferring mutations in global regulators against antibiotics.

10.1128/mSystems.00232-20.1 FIG S1 Schematic representation of the CREATE technology. The CREATE design software was used to design editing cassettes targeting different loci across the genome, and several such editing cassettes can be synthesized on DNA microarrays as pools. Subsequent high-throughput cloning and transformation into E. coli cells with active Cas9 and bacteriophage lambda Red recombination were used to construct large mutant libraries. Following selection, the initial and final library plasmids were performed by deep sequencing. Finally, log_2_-fold enrichment was used to calculate the fitness associated with all mutations in the library. Download FIG S1, TIF file, 2.8 MB. Copyright © 2020 Chen et al. 2020 Chen et al. This content is distributed under the terms of the Creative Commons Attribution 4.0 International license.

The activity of global regulations is extremely complex and spans multiple levels, ranging from the protein level to the regulon level. Therefore, studying a few mutations is not sufficient to understand their function. Although several proteins serve global regulatory and essential functions, only a few are frequently mutated in ALE (15). It is important to understand the distribution of the fitness effects of mutations in such genes. In order to address these challenges, we posited that a large library of mutations of global regulators would help rapidly identify mutations conferring fitness against diverse antibiotic stresses. Global regulators are mainly related to transcription and protein synthesis; therefore, the antibiotics used for selection are mainly protein synthesis inhibitors. We used CREATE to build a global regulatory library covering functional sites in global regulators. The library served as a platform for the expedited identification of previously uncharacterized mutations that confer fitness against doxycycline, thiamphenicol, gentamicin, or azithromycin stress. We also systematically studied the mechanism of genetic regulation of positive mutations using transcriptome analysis to explain the causes of antibiotic resistance.

## RESULTS

### Genome-wide global regulator library design.

Global regulators were selected by analysis of the literature (14, 24, 25) and databases, including the NCBI (https://www.ncbi.nlm.nih.gov/), Regulon DB (http://regulondb.ccg.unam.mx/), UniProt (https://www.uniprot.org/), and EcoCyc (https://ecocyc.org/) databases. As shown in Table 1, 23 genes in E. coli that are or that may be involved in global regulation were selected. Complete site saturation mutagenesis of all 23 global regulators would require 181,500 mutations, which was at the limit of the possible library size that could be obtained. Previous studies have highlighted that mutations in global regulatory proteins that enable adaptation occur predominantly in their functional regions (1). Therefore, in order to reduce the sequence space, we decided to target known and predicted functional residues. However, it was challenging to rapidly identify functional residues for two reasons. First, functional annotations of residues are dispersed across several databases, requiring significant effort to merge these data into a comprehensive set of targets. Second, the level of characterization was significantly variable across proteins. Therefore, we developed a pipeline to expedite the identification of functional residues.

**TABLE 1:** Global regulator libraries

Library name	Site type	Genes	Library size
G1	Active sites	*hns*, *cspA*, *arcA*, *fur*, *narL*, *lrp*, *mLc*, *ihfB*, *cspE*, *crp*, *phoB*, *dnaA*, *rpoS*, *rpoE*, *fnr*, *soxR*, *ihfA*, *fis*	7,000
G2	DNA binding sites	*cspA*, *arcA*, *narL*, *lrp*, *mLc*, *ihfB*, *cspE*, *argP*, *crp*	7,340
G3	DNA binding sites	*phoB*, *cytR*, *dnaA*, *soxS*, *rpoS*, *rpoN*, *rpoE*, *fnr*, *soxR*, *ihfA*, *fis*	7,400
G4	Dimerization	*arcA*, *fur*, *narL*, *mLc*, *ihfB*, *crp*, *phoB*, *cytR*, *rpoE*, *soxR*, *ihfA*, *fis*	5,260
G5	Predicted	*hns*, *cspA*, *arcA*, *fur*, *narL*, *lrp*, *mLc*, *cspE*, *argP*, *crp*, *phoB*, *cytR*, *dnaA*, *soxS*, *rpoS*, *fnr*, *soxR*, *ihfA*, *fis*, *rpoH*	7,340

The pipeline contained three modules. The first module used the protein sequence as input and then used blast analysis to identify available records in the NCBI, Regulon DB, UniProt, and EcoCyc databases. Next, we utilized custom Python scripts to predetermined and predicted functional residues from these records (26). The second module pulled the available structures from the Protein Data Bank (PDB) database. For ligand-bound structures, we used the PyMOL program to identify residues within a 5-Å radius from potential ligands (27). Finally, for the proteins without resolved protein structures, we used the iTasser suite of algorithms to predict their structure and potential ligand binding residues (28–30). Using a combination of these tools, we identified 1,700 residues across the 23 target proteins that covered over 30% of the possible amino acid sequence space of these proteins (Table 1). We then used the CREATE design software to design editing cassettes for 34,340 variants (23) (see Table S1 in the supplemental material). Since all the mutations would be difficult to cover within the same library, we divided the library into five sublibraries, libraries G1 to G5, with residues across proteins being categorized based on their function (Table 1). Subsequently, these cassettes were amplified and cloned into cells expressing Streptococcus pyogenes Cas9 and bacteriophage lambda Red recombineering proteins to generate the libraries. A major limitation of CRISPR-based high-throughput technologies is that differences in gRNA activity lead to a low overall editing efficiency of ∼1 to 2% at the library scale (31). Therefore, we plated and scraped 30- to 200-fold more colonies than the theoretical size of each sublibrary in order to ensure the efficient coverage of all mutations (Table S2).

10.1128/mSystems.00232-20.3 TABLE S1 Details of global regulatory libraries. Download Table S1, XLSX file, 0.02 MB. Copyright © 2020 Chen et al. 2020 Chen et al. This content is distributed under the terms of the Creative Commons Attribution 4.0 International license.

10.1128/mSystems.00232-20.4 TABLE S2 Transformation efficiency of the libraries. Download Table S2, DOCX file, 0.02 MB. Copyright © 2020 Chen et al. 2020 Chen et al. This content is distributed under the terms of the Creative Commons Attribution 4.0 International license.

### The global regulator library helped identify mutations that confer resistance to ribosome-targeting antibiotics.

**FIG 1:**
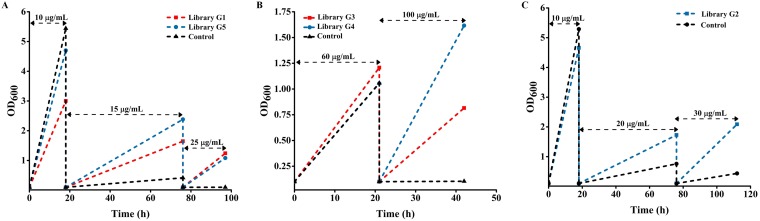
Library tolerance to the different antibiotics. (A) The mixed culture was used for inoculation of LB medium with doxycycline at 10 μg/ml, and then the resulting culture was transferred into fresh LB medium with doxycycline at a concentration of 15 μg/ml and then 25 μg/ml. (B) The mixed culture was used for inoculation of LB medium with 60-μg/ml thiamphenicol, and then the resulting culture was transferred into fresh LB medium with thiamphenicol at a concentration of 100 μg/ml. (C) The mixed culture was used for inoculation of LB medium with 10-μg/ml gentamicin, and then the resulting culture was transferred into fresh LB medium with gentamicin at a concentration of 20 μg/ml and then 30 μg/ml. OD_600_, optical density at 600 nm.

We performed selections using the global regulator library to identify mutations that improved tolerance to four ribosome-targeting antibiotics (protein synthesis inhibitors), including doxycycline, thiamphenicol, gentamicin, and azithromycin. The MIC for each antibiotic was first determined. The MICs of doxycycline, thiamphenicol, gentamicin, and azithromycin for E. coli were 25 μg/ml, 100 μg/ml, 30 μg/ml, and 120 μg/ml, respectively, in liquid lysogeny broth (LB) medium cultures. Cells can also gain resistance through natural evolution. Therefore, we performed selections over very short time scales within which natural resistance has not been reported previously. As shown in Fig. 1, we could complete the screening to obtain libraries with significant growth advantages via 2 to 3 transfers in the presence of different concentrations of antibiotics within 2 to 5 days.

For each antibiotic, we observed a significant growth advantage in one or more libraries (Fig. 1). However, the library, the duration, and the number of transfers in which the best resolution between these libraries and the control was achieved varied between antibiotics. For doxycycline, we observed an immediate growth advantage with the G1 and G5 libraries, and the libraries persisted to show high levels of resistance to a 25-μg/ml concentration (Fig. 1A). In the case of thiamphenicol and gentamicin, the nonedited control displayed growth profiles similar to those of the library with a low antibiotic concentration (Fig. 1B and C). In the case of thiamphenicol, a significant growth advantage was observed for the G3 and G4 libraries at 100 μg/ml of thiamphenicol (Fig. 1B). In the case of gentamicin, the G2 library demonstrated a significant growth benefit at a 30-μg/ml concentration (Fig. 1C), while for azithromycin, no library showed a significant growth advantage (data not shown). Therefore, the global regulator offered a platform to rapidly screen for variants that conferred resistance to different antibiotics.

In order to identify the fitness-conferring mutations, we used the editing cassette as a barcode to measure fitness using next-generation sequencing following the CREATE protocol (23). We identified mutations in the *soxR* and *crp* genes that had fitness scores significantly higher than those for the internal synonymous mutation controls (Table 2). We compared the list of positively enriched mutations against already known mutations for resistance to each antibiotic to classify a list of novel mutations for resistance to each antibiotic found in our study (Table 2).

**TABLE 2:** All potential mutations identified with altered amino acid sequence

Gene	Mutation	Fitness score^*a*^	Antibiotic
*soxR*	G121K	14.21059826	Doxycycline
*soxR*	I120E	13.41328904	Doxycycline
*soxR*	G121N	12.61816091	Doxycycline
*soxR*	G121P	10.68028162	Doxycycline
*soxR*	G121I	9.176806682	Doxycycline
*soxR*	I120D	2.9096309542	Doxycycline
*soxR*	I120E	1.3365831269	Thiamphenicol
*crp*	V140W	11.1088135691	Gentamicin

a The fitness score (or enrichment score) was used to evaluate the fitness contribution of each mutation under the various selection pressures investigated (23).

### Mutations in the global regulator *soxR* confer resistance to doxycycline, thiamphenicol, and azithromycin.

**FIG 2:**
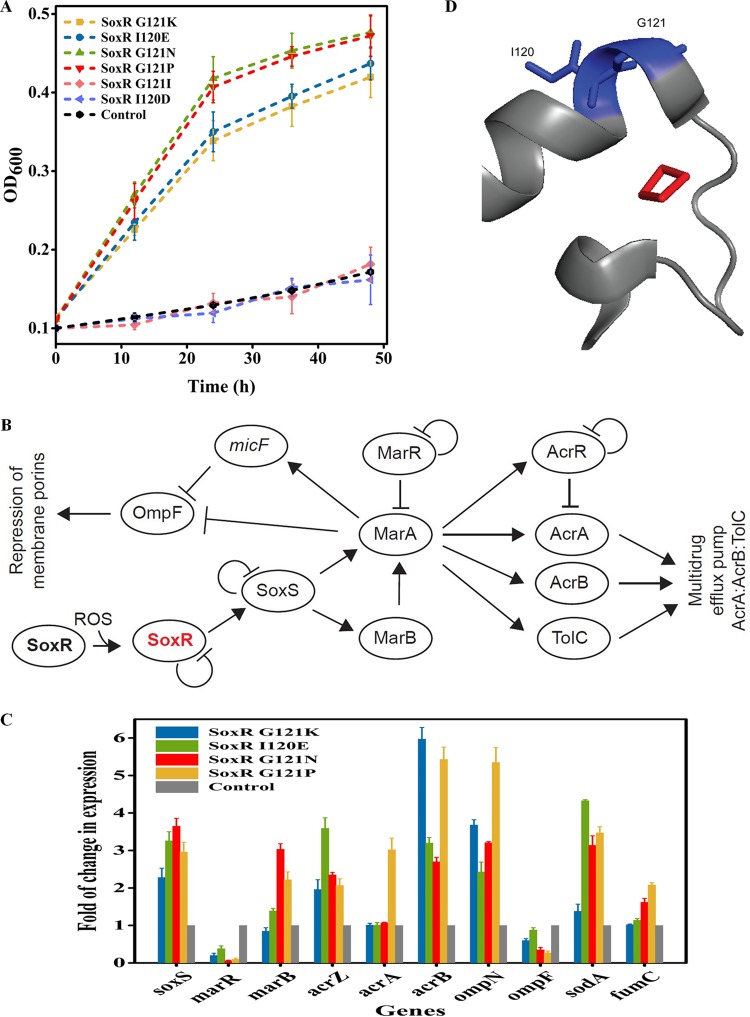
Mutations in SoxR confer resistance to doxycycline. (A) Verification of mutations enriched in the regulator library for resistance to doxycycline at a 25-μg/ml concentration. (B) The regulation network of SoxR control genes involved in multidrug efflux pumps and expression of porins. (C) Fold change in the levels of expression of SoxR-regulated genes in the SoxR G121K, I120E, G121N, and G121P mutants relative to their expression in strain MG1655 under a doxycycline concentration of 15 μg/ml. (D) The positions of mutated residues in the SoxR structure (blue). The ligand [2Fe-2S] is shown in red (PDB accession number 2ZHH).

We observed a significant enrichment of six novel mutations in the *soxR* gene for resistance to doxycycline (Table 2). We reconstructed the six mutations and compared the growth of the mutants with these mutations to that of wild-type strain MG1655 in the presence of 25-μg/ml doxycycline. Cells of four out of the six mutants, which had mutations in SoxR G121K, G121N, G121P, and I120E, had an improved growth rate compared to that of the wild-type cells (Fig. 2A). The mutants with two of the mutations, G121I and I120D, did not demonstrate significantly improved growth and the mutations were false positives, indicating that our read-mapping strategy may have incorrectly mapped some of the sequencing reads (Fig. 2A). The different mutations around the same cluster of targeted residues led to different levels of resistance against doxycycline, as determined by the differences in their growth rates (Fig. 2A).

SoxR is an integral component of the *soxRS* regulon, which regulates several genes for defense against reactive oxygen species (ROS) (32, 33). SoxR activates the transcription of the *soxS* gene in response to ROS, and SoxS increases the expression of antioxidant proteins and repair proteins (32). In order to short-list the possible target, we reconstructed the network of SoxR and SoxS to identify possible interactions with antibiotic resistance genes (Fig. 2B). The SoxS protein binds to the same DNA sequence as MarA, which activates the transcription of the *marRAB* genes (Fig. 2B). We found that a change in the expression of *marRAB* may improve tolerance to antibiotics by two separate pathways: increased expression of *acrAB*-*tolC* multidrug drug efflux pumps (34) and decreased expression of porins to reduce membrane permeability (35) (Fig. 2B).

In order to delineate the exact mechanism of resistance, we first used real-time (RT)-quantitative PCR (qPCR) to evaluate the change in expression of genes within the reconstructed network of SoxR. The network included the activator *soxS*; the genes *marR* and *marB*, which regulate multidrug efflux pumps and porins; efflux pump-related genes *acrZ*, *acrA*, and *acrB*; and membrane permeability-related genes (*ompN*, *ompF*). We also measured the changes in expression of the superoxide dismutase gene (*sodA*) and the tricarboxylic acid (TCA) cycle-related gene (*fumC*), which do not directly impact multidrug resistance, to understand the impact of the *soxR* mutation in the SoxRS regulon as well (Fig. 2C). We observed an increase in the expression of the *soxS* gene, suggesting that the mutations in SoxR likely reduced the repression capability of SoxR (Fig. 2C). Within *marRAB*, we observed a significant decrease in the expression of *marR* in all variants and an increase in the expression of *marB* in three out of four variants (Fig. 2C). While the expression of *acrB* and *acrA* increased significantly across variants, we observed a significant change in the level of expression of *acrA* for only one mutant, the G121P mutant (Fig. 2C). Meanwhile, the expression of the antibiotic-specific porin OmpF was reduced in the context of all of the mutants, while the expression of ion-related porin OmpN was increased compared to that in the wild-type control (Fig. 2C). The data showed that while a highly parallel response of changing trends in expression was observed across variants, the magnitude of the effect across these regulons for each mutation was variable.

The [2Fe-2S] cluster is essential for the activity of SoxR (36). Different ROS oxidize [2Fe-2S] to activate the expression of SoxR, which in turn activates the expression of SoxS. The oxidation of [2Fe-2S] leads to conformational changes to activate the SoxR protein and, consequently, SoxS. Both residues G121 and I120 occur within the same alpha helix adjacent to the C119 and C122 residues, which directly interact with [2Fe-2S] (37) (Fig. 2D). These mutations likely alter the binding of SoxR to [2Fe-2S], which constitutively activates SoxR. Consequently, as expected, we observed an increase in SoxS-regulated genes *sodA* and *fumC* in the ROS regulon (Fig. 2C).

**FIG 3:**
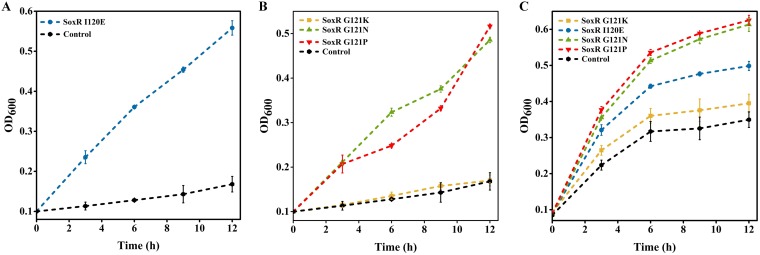
Mutations in SoxR are pleiotropic for resistance to different antibiotics. (A) Verification of the mutations enriched in the global regulator library for resistance to thiamphenicol at a 100-μg/ml concentration. (B) Evaluation of the extent of synergistic pleiotropy for the mutations in *soxR* conferring resistance to doxycycline for resistance to thiamphenicol at a 100-μg/ml concentration. (C) Evaluation of the extent of synergistic pleiotropy for all mutations in *soxR* conferring resistance to doxycycline for resistance to azithromycin at a 120-μg/ml concentration. The colored lines indicate the variants, and the black lines indicate wild-type E. coli.

Interestingly, one of the mutations in *soxR* found to confer resistance to doxycycline, I120E, also conferred resistance to thiamphenicol (Fig. 3A). As mentioned above, mutations in SoxR may lead to increased expression of efflux pumps and decreased expression of porins. The regulation of transport by porins is nonspecific (7, 8). The downregulation of porin expression in response to one drug may also significantly increase resistance to other, new drugs, enabling multidrug resistance (9–12). The AcrA-AcrB-TolC multidrug efflux pump is also nonspecific and has the ability to target a wide range of structurally dissimilar antibiotics, again facilitating multidrug resistance (6). Therefore, we next evaluated the extent of synergistic pleiotropy for the resistance-conferring mutations in *soxR* for resistance to doxycycline for resistance to other antibiotics. Mutants with the G121N and G121P mutations in the protein encoded by *soxR* were also found to be resistant to thiamphenicol (Fig. 3B). All mutants with mutations conferring resistance to thiamphenicol were also resistant to azithromycin (Fig. 3C). Therefore, mutants with several mutations in *soxR* demonstrated resistance to several different antibiotics.

### Genome-wide transcriptional changes in the SoxR G121P mutant in the presence of doxycycline.

**FIG 4:**
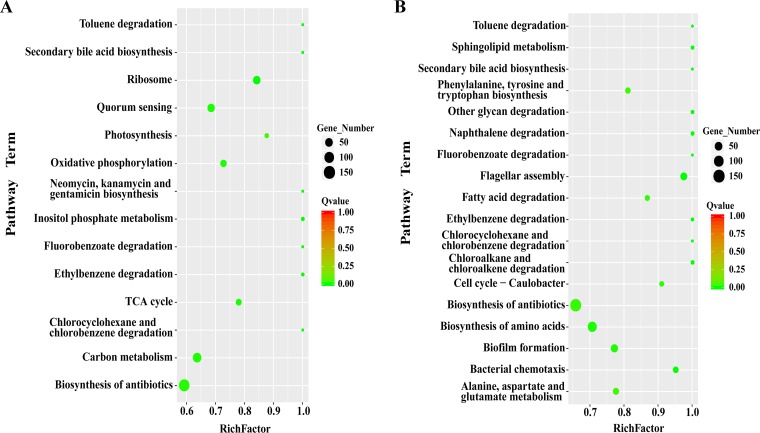
Scatterplot of unigenes mapped to the KEGG database. (A) Strain DC and the SoxR G121P mutant. (B) Strain GC and the CRP V140W mutant. Rich factor, the ratio of the number of genes with significant differences in transcription levels to the total number of all annotated genes in the pathway. *Q* value, the *P* value after correction by the multiple-hypothesis test.

SoxR is a global regulator that controls the expression of several genes in E. coli. Therefore, mutations in *soxR* could alter the expression of several genes. Such expression changes could also contribute to adaptation to doxycycline. In order to explore the expression changes and possible tolerance mechanisms, we evaluated the genome-wide expression changes, using transcriptome sequencing (RNA-seq), between the mutant strain with SoxR G121P and the control strain, E. coli MG1655 (strain DC), in the presence of 15 μg/ml doxycycline, which was below the MIC of doxycycline, to allow the growth of both wild-type and mutant cells. A summary of the detailed RNA-seq data is provided in Table S3. We identified that 48% of the genes in the strain with the SoxR G121P mutation were differentially expressed compared to their expression in the wild-type strain (fold change ≥ 2; adjusted *P* value ≤ 0.05) in the presence of doxycycline. Of these, the expression of 49% of the genes was upregulated and the expression of 51% of the genes was downregulated (Fig. S2). We analyzed the differentially expressed genes (DEGs) by use of the Kyoto Encyclopedia of Genes and Genomes (KEGG) and found that the expression of the genes in 14 pathways changed significantly with a *Q*-value threshold of <0.05 in the presence of doxycycline (Fig. 4A). Of these pathways, the ribosome synthesis pathway, the TCA cycle, oxidative phosphorylation, carbon metabolism, and antibiotic biosynthesis may affect the adaptation of the cells to doxycycline. Therefore, we further validated the changes in the expression of genes for ribosome synthesis, carbohydrate metabolism, and oxidative phosphorylation by confirming the expression changes using RT-qPCR (Table 3).

**TABLE 3:** Relative expression levels of 23 genes affected by SoxR G121P in response to doxycycline

Gene	Name of gene product	Fold change in expression determined by:	
		RNA-seq	Real-time qPCR
*rpsR*	30S ribosomal subunit protein S18	−25.93	−166.96
*rplD*	50S ribosomal subunit protein L4	−20.44	−85.04
*rpsF*	30S ribosomal subunit protein S6	−18.45	−156.14
*rpsC*	30S ribosomal subunit protein S3	−16.13	−9.04
*rpsQ*	30S ribosomal subunit protein S17	−8.16	−83.87
*rpsB*	30S ribosomal subunit protein S2	−6.59	−60.13
*rplE*	50S ribosomal subunit protein L5	−3.90	−23.53
*fbaB*	Fructose-bisphosphate aldolase class I	106.86	15.60
*pgi*	Phosphohexose isomerase	10.24	1.24
*pfkA*	6-Phosphofructokinase I	−44.20	−2,586.29
*aceE*	Pyruvate dehydrogenase	−3.82	−20.16
*talA*	Transaldolase A	80.72	13.06
*tktB*	Transketolase B	60.17	11.82
*fumC*	Fumarate hydratase	76.81	7.52
*sucD*	Succinyl-CoA synthetase	17.50	1.75
*acnA*	Aconitate hydratase	17.49	2.44
*sucA*	2-Oxoglutarate decarboxylase	14.18	1.69
*mqo*	Malate dehydrogenase	−15.55	−7.78
*aceA*	Isocitrate lyase	13.08	1.47
*aceB*	Malate synthase A	12.00	1.53
*ndh*	NADH dehydrogenase II	−3.66	−4.82
*gadB*	Glutamate decarboxylase	873.93	33.98
*gadA*	Glutamate decarboxylase	355.04	8.22

10.1128/mSystems.00232-20.2 FIG S2 Number of genes differentially expressed in the SoxR G121P and CRP V140W mutants. The gray, red, and blue columns are the number of total differentially expressed genes (DEGs), upregulated DEGs, and downregulated DEGs, respectively. Download FIG S2, TIF file, 0.3 MB. Copyright © 2020 Chen et al. 2020 Chen et al. This content is distributed under the terms of the Creative Commons Attribution 4.0 International license.

10.1128/mSystems.00232-20.5 TABLE S3 Summary of RNA-seq data. Download Table S3, DOCX file, 0.01 MB. Copyright © 2020 Chen et al. 2020 Chen et al. This content is distributed under the terms of the Creative Commons Attribution 4.0 International license.

**FIG 5:**
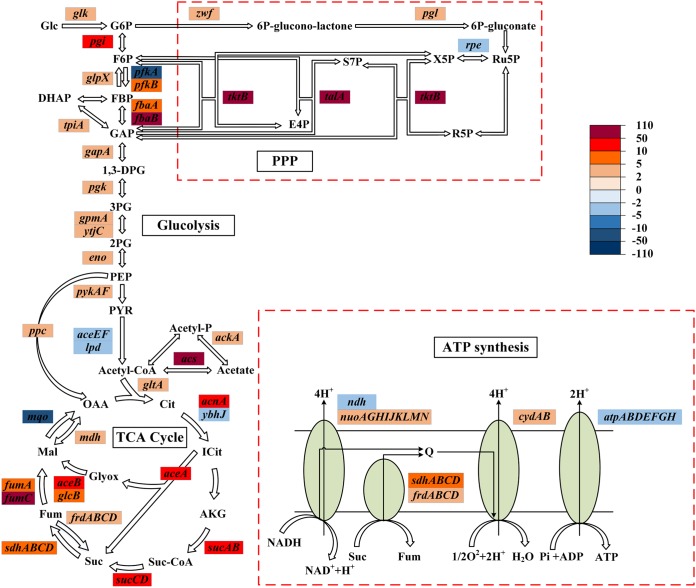
Heat map of the expression levels of genes for carbohydrate metabolism and oxidative phosphorylation in the SoxR G121P mutant. The fold change in expression for DEGs is represented by the color code on the heat map. The red code indicates that the gene was upregulated, and the blue code indicates that the gene was downregulated. Glc, glucose; G6P, glucose-6-phosphate; F6P, fructose-6-phosphate; FBP, fructose-1,6-bisphosphate; DHAP, dihydroxyacetone phosphate; GAP, glyceraldehyde-3-phosphate; 1,3-DPG, 1,3-bisphosphoglycerate; 3PG, 3-phosphoglycerate; 2PG, 2-phosphoglycerate; PEP, phosphoenol pyruvate; PYR, pyruvate; Acetyl-CoA, acetyl coenzyme A; Cit, citrate; ICit, isocitric acid; AKG, oxoglutarate; Suc-CoA, succinyl coenzyme A; Suc, succinic acid; Fum, fumarate; Mal, malic acid; Glyox, glyoxylate; OAA, oxaloacetate; Ru5P, ribulose-5-phosphate; X5P, xylulose-5-phosphate; R5P, ribose-5-phosphate; E4P, erythrose-4-phosphate; S7P, seduheptulose-7-phosphate; PPP, pentose phosphate pathway.

In the SoxR G121P mutant, we observed an increase in the expression of 81% of the genes involved in carbohydrate metabolism (Table S4). We found that several DEGs in these pathways were upregulated, particularly genes involved in glycolysis (*pgi*, *pfkB*, *fbaAB*), the pentose phosphate pathway (*tktB*, *talA*), the TCA cycle (*acnA*, *sucABCD*, *sdhABCD*, *fumAC*), the glyoxylate cycle (*aceAB*, *glcB*), and oxidative phosphorylation (*nuoAGHIJKLMN*, *sdhABCD*, *frdABCD*, and *cydAB*) (Fig. 5). We confirmed the increase in the expression of the genes *fbaB*, *pgi*, *talA*, *tktB*, *fumC*, *sucAD*, *acnA*, and *aceAB* using RT-qPCR (Table 3). However, the levels of expression of the *atp* operon, which encodes ATP synthase, and 87% of the ribosomal genes were reduced. The mutation G121P in the global regulatory factor SoxR altered the expression level of genes involved in carbohydrate and energy metabolism, which is likely to improve the production of NADPH and NADH/ATP and further promote cell growth for improved adaptation to the doxycycline stress.

10.1128/mSystems.00232-20.6 TABLE S4 Relative expression levels of genes involved in ribosome synthesis, carbohydrate metabolism, and oxidative phosphorylation affected by SoxR G121P in response to doxycycline. Download Table S4, DOCX file, 0.03 MB. Copyright © 2020 Chen et al. 2020 Chen et al. This content is distributed under the terms of the Creative Commons Attribution 4.0 International license.

### A mutation in the *crp* gene confers resistance to gentamicin and azithromycin.

**FIG 6:**
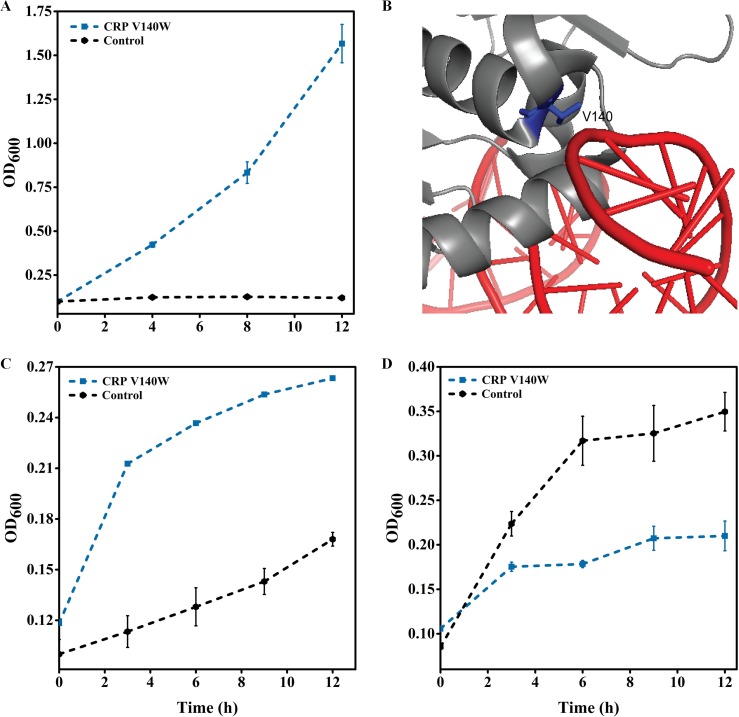
Mutations in CRP confer resistance to gentamicin and are pleiotropic for resistance to other antibiotics. (A) Verification of mutations enriched in the regulator library for resistance to gentamicin at a 30-μg/ml concentration. (B) Position of mutated residues (blue) on the CRP structure (gray) and their proximity to DNA (red). (C) Evaluation of the extent of synergistic pleiotropy for the mutation in *crp* conferring resistance to gentamicin for resistance to thiamphenicol at a 100-μg/ml concentration. (D) Evaluation of the extent of synergistic pleiotropy for the mutation in *crp* conferring resistance to gentamicin for resistance to azithromycin at a 120-μg/ml concentration. The blue lines indicate variants, and the black lines indicate wild-type E. coli. All strains used to verify resistance and pleiotropy were cultured in LB medium (10-g/liter tryptone, 5-g/liter yeast extract, 10-g/liter NaCl) with the corresponding antibiotic at 37°C and 220 rpm in 250-ml conical flasks with a 50-ml final volume of the medium.

We found that a mutation in the protein encoding the *crp* gene, V140W, was enriched in the selections against gentamicin. We reconstructed this mutation and compared the growth of the mutant to that of the wild-type strain in 30-μg/ml gentamicin. Upon reconstruction, this enriched variant had significantly improved growth in the presence of gentamicin compared to that of the wild-type strain (Fig. 6A). Residue V140 occurs in the DNA binding C-terminal domain of the cAMP receptor protein (CRP) (38) (Fig. 6B). The mutation possibly changes the hydrogen bond between V140 and the DNA chain to change the strength of CRP-DNA binding and alter expression (39).

CRP is a master global regulator that controls the expression of over 490 genes in E. coli (40). Therefore, like *soxR*, we posited that mutation of the gene for this protein may also be pleiotropic to increase resistance to other drugs. So, we grew the strain with this mutation in azithromycin and thiamphenicol. The strain demonstrated improved growth in the presence of thiamphenicol compared to that of the control (Fig. 6C). It had reduced growth in azithromycin compared to that of the control (Fig. 6D). Therefore, the mutation V140W was pleotropic and altered the resistance of E. coli to gentamicin and azithromycin.

### Genome-wide transcriptional changes in the CRP V140W mutant under gentamicin stress.

CRP is a global transcriptional regulator that controls the expression of over 400 genes involved in carbon metabolism, biofilm formation, ion uptake, antibiotic multidrug resistance, and other functions (40, 41). CRP can also directly or indirectly activate the redox-sensing regulator SoxR to regulate *soxS* gene in response to ROS (42). Additionally, CRP can also activate *marRAB* gene expression, which can activate the AcrA-AcrB-TolC multidrug efflux pump, as mentioned before (43). Using RNA-seq of the strain with the mutation CRP V140W, we identified that 48% of the genes were differentially expressed in strain CRP V140W compared to their expression in the wild-type strain (strain GC) in the presence of 5-μg/ml gentamicin. Of these, 44% of the genes were upregulated and 56% of the genes were downregulated in the mutant compared to their expression in GC (Fig. S2). Based on KEGG analysis, 19 pathways were enriched in the mutant in the presence of gentamicin (Fig. 4B). Of the genes for these pathways, genes involved in the biosynthesis of amino acids, fatty acid degradation, and the TCA cycle may affect the adaptation of the cells to gentamicin. Therefore, we validated the expression of genes involved in the biosynthesis of amino acids and fatty acid degradation using RT-qPCR (Table 4).

**TABLE 4:** Relative expression levels of 21 genes affected by CRP V140W in response to gentamicin

Gene	Name of gene product	Fold change in expression determined by:	
		RNA-seq	Real-time qPCR
*flgB*	Flagellar basal body rod protein	−308.10	−747.88
*fliA*	RNA polymerase sigma factor for flagellar operon	−213.54	−429.55
*motA*	Chemotaxis protein	−138.11	−592.22
*motB*	Chemotaxis protein	−74.83	−342.51
*flhD*	Flagellar class II regulon transcriptional activator	−19.34	−75.41
*flhC*	Flagellar class II regulon transcriptional activator	−13.43	−92.20
*tar*	Methyl-accepting chemotaxis protein II	−164.92	−403.57
*cheA*	Two-component system, chemotaxis family, sensor kinase	−44.62	−327.04
*pdeH*	Cyclic-di-GMP phosphodiesterase	−107.17	−367.94
*flu*	CP4-44 prophage antigen 43 phase-variable biofilm formation autotransporter	−12.74	−45.78
*leuB*	3-Isopropylmalate dehydrogenase	41.74	1.64
*leuA*	2-Isopropylmalate synthase	40.36	1.31
*serC*	3-Phosphoserine/phosphohydroxythreonine aminotransferase	8.28	1.30
*tdcB*	l-Threonine dehydratase	−448.77	−11,036.54
*fadB*	3-Hydroxyacyl-CoA dehydrogenase/enoyl-CoA hydratase/3-hydroxybutyryl-CoA epimerase/enoyl-CoA isomerase	−21.58	−167.73
*yqeF*	Beta-ketoacyl-CoA thiolase	−6.72	−52.35
*tktB*	Transketolase B	36.99	3.46
*talA*	Transaldolase A	28.78	5.46
*pfkA*	6-Phosphofructokinase I	−89.48	−8,079.22
*sdhC*	Succinate dehydrogenase	−27.10	−307.26
*mqo*	Malate dehydrogenase	−6.49	−73.18

We found that 83 DEGs were clustered in amino acid transport and metabolism (Table S5). Of these, genes involved in histidine, valine, leucine, isoleucine, tryptophan, tyrosine, and phenylalanine were upregulated (Table S5). The increase in expression was the most significant in the *leuABCD* genes, involved in leucine biosynthesis. Using RT-qPCR, we found that the expression of the *leuA* and *leuB* genes increased 1.31-fold and 1.64-fold, respectively (Table 4). Therefore, the fold change in gene expression found using RNA-seq corroborated the expression change found using RT-qPCR. We also observed an increase in the expression of genes involved in serine biosynthesis and confirmed the increase in expression of the *serC* gene, involved in the serine biosynthesis pathway (Table 4). On the contrary, we observed a decrease in the expression of genes involved in amino acid metabolism (tyrosine, serine, cysteine, methionine, and arginine) and fatty acid degradation (*fadABDEIJ* and *yqeF*) (Table S5). As a confirmation, the *tdcB* gene, involved in the serine metabolism pathway, showed the highest level of downregulation, as determined using both RNA-seq and RT-qPCR (Table 4). The mutation V140W in CRP altered the expression of genes involved in amino acid transport and fatty acid degradation, which may reduce the cell proton motive force (PMF) and reduce the formation of acetyl coenzyme A (CoA), NADH, and FADH_2_, which further alter the cellular energy state to improve gentamicin tolerance.

10.1128/mSystems.00232-20.7 TABLE S5 Relative expression levels of genes involved in biosynthesis of amino acids and fatty acid degradation affected by CRP V140W in response to gentamicin. Download Table S5, DOCX file, 0.03 MB. Copyright © 2020 Chen et al. 2020 Chen et al. This content is distributed under the terms of the Creative Commons Attribution 4.0 International license.

## DISCUSSION

As we proposed, using the global regulator library, we were able to identify mutations that improved tolerance to the stress induced by a diversity of ribosome-targeting antibiotics, including doxycycline, thiamphenicol, and gentamicin. What is more, we could complete screening and mapping in 1 to 2 weeks to identify mutations that appear under conditions of antibiotic stress. Several interesting trends in pleiotropy also emerged in our data. We identified 1 to 4 positive hits across different antibiotics. Interestingly, mutants with three mutations in *soxR* which improved tolerance to doxycycline were also found to be resistant to thiamphenicol. However, a mutation in *crp* also conferred resistance to the antibiotics gentamicin and chloramphenicol (a chemical variant of thiamphenicol). This finding highlights the suggestion that the expression of nonspecific resistance-conferring pathways is controlled by these global regulators. Multisite mutations may have composite effects, but the limitations of the CREATE technology prevented us from obtaining combinations of mutations at multiple loci, which is extremely important for the study of antibiotic resistance. Therefore, future studies will investigate the effect of combinations of mutations at multiple loci on antibiotic resistance. Further rounds of CREATE in an iterative fashion with some of these mutations might be useful to further define the combinatorial mutational landscapes of antibiotic resistance phenotypes.

An in-depth analysis of the transcriptome in different environments provided significant input on the tolerance to antibiotics and the possible molecular bases of cross-resistance. Genome-wide expression analysis using RNA-seq helped us understand the adaptive changes in the SoxR G121P mutant compared to wild-type E. coli. First, we observed an increase in the expression of glycolytic genes and pentose phosphate pathway genes. We postulate that the increase in the expression of these genes may help improve glucose utilization to produce more ATP/NADH in the mutant strain than in the wild-type strain. The increase in expression of these genes may also improve the production of NADPH to improve the cell’s capability for biosynthesis. The improved biosynthesis hypothesis was further corroborated by the observed increase in the expression of TCA cycle genes, which provide precursors for the synthesis of amino acids and other small molecules (44). We also observed an increased expression of oxidative phosphorylation genes, which may have improved ATP production to promote improved cell growth in the SoxR G121P mutant compared to the wild type.

Interestingly, in the SoxR G121P mutant, the expression of ATP synthase, encoded by the *atp* operon, was slightly reduced, probably because proton leakage may change the proton gradient to inhibit the expression of ATP synthase to a certain extent (45, 46). In addition, the intake of doxycycline requires the PMF to provide energy (44, 47), and DEGs involved in the respiratory chain may prevent the intake of doxycycline, thus improving the tolerance of E. coli to doxycycline.

Doxycycline binds to the tRNA binding sites (A site) of the 30S small subunit in the ribosome (48), which downregulates the expression of the *rpsR*, *rplD*, *rpsF*, *rpsC*, *rpsQ*, *rpsB*, and *rplE* genes, involved in ribosome biosynthesis (Table 3). The expression of these genes likely decreased in the mutant because of the decrease in the intracellular doxycycline concentration as a result of the altered expression of efflux pumps, as mentioned above. The reduced intracellular levels of doxycycline may have partially relieved the inhibition of peptide chain elongation and promoted protein synthesis. Therefore, genome-wide expression analysis allowed us to capture adaptive changes in the SoxR G121P mutant in response to improved tolerance to doxycycline.

The mutation in CRP improved the adaptation to gentamicin by global rewiring of cellular metabolism, particularly amino acid metabolism and degradation. A study by Shan et al. reported that the deletion of amino acid synthesis genes increased the sensitivity of E. coli to gentamicin (44). However, interestingly, they found that supplementation of the growth medium of a specific amino acid auxotroph with amino acids did not improve tolerance to gentamicin. The authors posited that the increase in the synthesis of amino acids changes the energy state of the cells. Gentamicin uptake in cells requires proton pumps; thus, it is proposed that increased biosynthesis may reduce the cell proton motive force to improve gentamicin tolerance. To confirm this hypothesis, in addition to increased biosynthesis, we also observed that genes involved in tyrosine, serine, cysteine, methionine, and arginine metabolism were downregulated (see Table S5 in the supplemental material), which could further alter the cellular energy state to improve gentamicin tolerance. Furthermore, we also observed a decrease in fatty acid degradation genes (*fadABDEIJ* and *yqeF*), which would further reduce the level of formation of acetyl-CoA, NADH, and FADH_2_ and change the cellular energy state. Therefore, a mutation in CRP, the master metabolic regulator, may have provided improved tolerance to gentamicin by altering the cellular energy state.

The positive mutations identified in *soxR* and *crp* were pleiotropic. As discussed above, we observed that mutations in SoxR led to the increased expression of efflux pumps and the decreased expression of porins (Fig. 2C). The regulation of transport by porins is nonspecific (7, 8). The downregulation of porin expression in response to one drug may significantly also increase resistance to other, new drugs, enabling multidrug resistance (9–12). The AcrA-AcrB-TolC multidrug efflux pump, whose expression changed in the SoxR mutants, is also nonspecific and has the ability to target a wide range of structurally dissimilar antibiotics, again facilitating multidrug resistance (2). Therefore, a mutant with mutations in SoxR demonstrated increased tolerance to diverse antibiotics (Fig. 3B). Similarly, a mutation in CRP that resulted in improved tolerance to gentamicin also improved tolerance to thiamphenicol. As discussed above, the mutation in CRP altered the cellular energy state, which could alter the proton motive force for drug efflux. As in the SoxR mutant, the nonspecificity of drug efflux may have rendered the CRP mutant pleiotropic as well.

In summary, we developed the global regulator library in E. coli, which served as a platform to expedite the identification of novel mutations that confer tolerance to doxycycline, thiamphenicol, and gentamicin. Then, we evaluated the genome-wide changes in gene expression in E. coli for two novel mutations under conditions of doxycycline or gentamicin stress. The method used in this study has given us a better understanding of the mechanism of resistance to doxycycline and gentamicin and also provides a method for quickly identifying resistance-related mutations. In addition to antibiotic resistance, this method can also be used to study the tolerance of chemicals, organic solvents, and oxidative stress. This method can be further combined with the proteome to understand such nonspecific mechanisms of resistance and to identify combinations of treatments or conditions that may slow adaptive evolution. It may be key to developing new antibacterial drugs, monitoring potential drug-resistant strains, controlling environmental pollution caused by antibiotics, and strengthening food safety.

## MATERIALS AND METHODS

### Library construction.

We used the CREATE technology to develop libraries designed in E. coli (23). We designed a unique editing cassette corresponding to each targeted mutation, and the cassettes were synthesized on microarray chips. Subsequently, we used Phusion Q5 polymerase (New England Biolabs [NEB]) to amplify all libraries from the purified microarray chip and plasmid backbones (23). PCR was performed with 2.5 μl each of 10 μM forward and reverse primers, 1 μl of template (∼1 to 10 ng/μl), 25 μl of NEB Q5 2× master mix and 19 μl of nuclease-free double-distilled water under standard PCR conditions for HF Phusion polymerase (98°C for 30 s; 34 cycles of 98°C for 15 s, the melting temperature [*T_m_*] for 15 s, and 72°C for 15 s × [length of amplicon]; and 1 cycle of 72°C for 5 min). Primer *T_m_* values were calculated using the NEB *T_m_* calculator. For all plasmid backbones, the amplification was followed by a DpnI digestion reaction; 1 μl of DpnI (NEB) was added to the PCR amplification reaction mixture, and the solution was incubated at 37°C for 1 h. The amplicons were purified by gel extraction using a Qiagen gel extraction kit. The inserts were cloned into plasmids using circular polymerase extension cloning (CPEC) with 12.5 μl of an equimolar mixture of the insert and the backbone with at least 100 ng of backbone and 12.5 μl of 2× HF Phusion master mix (NEB). PCRs were carried out at 98°C for 30 s, followed by 10 cycles of 98°C for 10 s, 55°C for 10 s, and 72°C for 90 s and then 72°C for 120 s, followed by a hold at 12°C. Ten microliters of the CPEC reaction mixture was transformed into competent cells by electroporation. The transformed cells were plated on LB medium with 100-μg/ml spectinomycin.

### Cas9-mediated bacteriophage lambda Red recombineering.

All editing was performed in E. coli MG1655 (a K-12 strain). The bacteriophage lambda Red recombineering protocol was followed, using cells that had been previously transformed with either the pX2cas9 or the proN-X2cas9 plasmid and the pSIM5 bacteriophage lambda recombination plasmid (49). Starter cultures of cells containing the pSIM5 recombineering plasmid and the plasmid expressing *cas9* were grown in LB containing 0.4% arabinose, 40-μg/ml kanamycin, and 40-μg/ml chloramphenicol at 30°C overnight. The starter cultures were diluted 1:100 into 50- to 250-ml cultures and grown to mid-exponential phase (optical density at 600 nm, 0.35 to 0.4). The cells were incubated at 42°C for 15 min to induce bacteriophage lambda Red recombination proteins and then cooled on ice for 15 to 20 min. Aliquots (45 ml) were subjected to centrifugation at 7,500 × *g* at 4°C for 3 min. The supernatant was discarded, and the pellets were washed in 25 ml ice-cold sterilized double-distilled water by resuspending and centrifuging at 7,500 × *g* at 4°C for 3 min. The cells were washed thrice in prechilled (4°C) water and once in prechilled (4°C) 10% glycerol. Finally, the cells were resuspended in a volume of 10% glycerol 100-fold lower than the culture volume. For editing, cells were transformed either with the library plasmids (or editing plasmids) or with the guide RNA plasmid and the linear homology repair template in 0.1-mm electroporation cuvettes. Transformed cells were allowed to recover for 3 h in either LB or LB with 0.4% arabinose when the *araC* promoter was used to control the expression of *cas9*.

### Antibiotic selections.

For each antibiotic, we first made a 1:100 dilution for overnight cultures of each library and control population in LB containing the antibiotic at less than one-fourth of the MIC. After library construction, we waited until the libraries grew to the saturation optical density and transferred the cells into LB medium with subinhibitory concentrations of antibiotics. In the subsequent transfer, we increased the dosage of the antibiotics. We repeated this transfer until we observed a significant growth advantage for the libraries over the control populations.

### CREATE deep sequencing and fitness calculation.

In the CREATE technology, the mutations are tracked using the editing cassettes as barcodes. For the deep sequencing experiments, the plasmids were extracted from cell samples stored before and selected using a standard plasmid miniprep kit (50). Following extraction, PCR was performed using custom CREATE next-generation sequencing primers and NEB Q5 polymerase following the protocol discussed above. In the first PCR step, a unique barcode was ligated for each experimental sample. After PCR, the samples were extracted using gel purification. Samples from different experiments were then pooled into an equimolar mixture. Finally, a second PCR was carried out to attach the Illumina next-generation sequencing adapters to the 1st PCR product. All sequencing was performed using a Nextera next-generation sequencing kit with an Illumina sequencer (50).

In order to identify the fitness-conferring mutations, the editing cassette was used to quantify the fitness associated with different mutations in our library. Then, log_2_-fold enrichment was used to calculate the fitness associated with all mutations in our library. In each library, synonymous mutations for each position were a part of each library. The synonymous mutations were used to determine the mean and standard deviation (SD) of the log_2_ fitness change of wild-type cells. Then, a mean + 2× SD cutoff was used to estimate strongly enriched mutations in the selections.

### Reconstruction of mutant strains.

To construct the gRNA plasmids, the pGRB backbones were first amplified from the pGRB plasmid using the primers pGRS-F/R and pGRC-F/R. Then, the pGRB backbones were self-ligated to create gRNA plasmids pGRS and pGRC. To construct donor double-stranded DNA (dsDNA), upstream and downstream homologous arms were amplified from the E. coli MG1655 genome, and then these were fused together (51). The mutant strains were obtained by cotransforming gRNA plasmids and exogenous donor dsDNA into competent cells containing the pTKREDCas9 plasmid by means of the CRISPR genome editing system (51). By this method, E. coli MG1655 strains with SoxR G121K, SoxR 1120E, SoxR G121N, SoxR G121P, SoxR G121I, SoxR G121D, or CRP V140W were obtained. Details of the protocol of variant construction are provided in reference 51. All primers used for the reconstruction of mutant strains are listed in Table S6 in the supplemental material.

10.1128/mSystems.00232-20.8 TABLE S6 Primers used for reconstruction of mutant strains. Download Table S6, DOCX file, 0.02 MB. Copyright © 2020 Chen et al. 2020 Chen et al. This content is distributed under the terms of the Creative Commons Attribution 4.0 International license.

### RNA-seq-based transcriptome analyses.

Using an Illumina HiSeq sequencer, RNA sequencing was performed on four strains, including the SoxR G121P mutant strain and its control, E. coli MG1655, and the CRP V140W mutant strain and its control, E. coli MG1655. Each strain was tested in three independent replicates. Total RNA was extracted from the samples with an RNeasy minikit (Qiagen, Valencia, CA, USA) after the cells of positive variant strains and control strains were grown to mid-exponential phase at 37°C and 220 rpm. The quantity and quality of RNA were verified using a Bioanalyzer system (Agilent Technologies, Palo Alto, CA, USA). Total RNA (1 μg) with an RNA integrity number (RIN) value above 6.5 was used for following library preparation. Next-generation sequencing library preparations were constructed according to the manufacturer’s protocol (NEBNext Ultra RNA library preparation kit for the Illumina sequencer; NEB). The rRNA was depleted from total RNA using an rRNA removal kit. The rRNA-depleted RNA was then fragmented and reverse transcribed to synthesize cDNA. Then, 12 independent libraries with different indices were multiplexed and loaded on an Illumina HiSeq instrument according to the manufacturer’s instructions (Illumina, San Diego, CA, USA). Sequencing was carried out using a 2 × 150 paired-end (PE) configuration. Feature-extracted raw data in the fastq format were analyzed using the Trimmomatic (v0.30) tool to remove technical sequences. First, the transcripts in the fastq format were converted from a known .gff annotation file and indexed properly. Then, the HTSeq (v0.6.1p1) package was used to estimate gene expression levels from the paired-end clean data (52). Using the DESeq2 Bioconductor package, which is a model based on the negative binomial distribution, differential expression analysis was conducted. Differentially expressed genes were detected based on a *P* value of <0.05, after adjustment by the Benjamini and Hochberg approach for controlling the false discovery rate (53). The Kyoto Encyclopedia of Genes and Genomes (KEGG) is a collection of databases dealing with genomes, biological pathways, diseases, drugs, and chemical substances (http://en.wikipedia.org/wiki/KEGG). We used scripts developed in-house to detect genes in KEGG pathways that were significantly differently expressed (*Q* value ≤ 0.05). The *Q* value is determined by correction of the *P* value by a multiple-hypothesis test.

### Real-time qPCR.

Total RNA was extracted from each sample with an RNeasy minikit (Qiagen, Valencia, CA, USA), after the cells of positive variant strains were grown to mid-exponential phase at 37°C and 220 rpm. cDNA synthesis was performed with TransScript first-strand cDNA synthesis SuperMix (Transgen Corp., Beijing, China) with random primers, which achieved reverse transcription of total RNA. After that, real-time qPCR of targeted genes from test strains was performed using TransStart Top Green qPCR SuperMix (Transgen Corp., Beijing, China) and a LightCycler 480 device (Roche, Switzerland). To ensure accuracy, the 16S RNA of E. coli was used as an internal standard control to normalize the transcript levels (54). The primers used in RT-qPCR are listed in Table S7. Triple replicates were used both for each targeted gene and for the test strains.

10.1128/mSystems.00232-20.9 TABLE S7 Primers used for RT-qPCR. Download Table S7, DOCX file, 0.02 MB. Copyright © 2020 Chen et al. 2020 Chen et al. This content is distributed under the terms of the Creative Commons Attribution 4.0 International license.

### Data analysis.

An analysis pipeline for estimating the number of reads with the targeted genomic edit was developed. The initial assembly of forward and reverse reads was performed using the PandaSeq tool (55) and the USEARCH algorithm (56). Experimental barcodes were split using our analysis code. Reads were mapped to their mutant genotypes using the USEARCH algorithm (56), with correctly mapped reads having >98.3% identity to the target sequence, allowing an error of 3 mismatches over an ∼450-bp sequenced region. The actual editing efficiencies were calculated as the ratio of the read counts mapped to the mutant phenotype to the total number of reads obtained for the sample. In order to map base change frequencies, the reads were compared to the wild-type sequence with a 95% identity threshold using the USEARCH algorithm (56). We developed code to use identity/mismatch mapping to calculate the frequency of mismatches at each position. The base change frequency was the ratio of the total number of reads with the base changed to the total number of reads with perfect (100%) identity to the wild type. Scipy and Numpy kits from Python were used for all data analyses.

### Data availability.

The RNA sequencing data have been submitted to the NCBI BioProject database with accession number PRJNA602113.
