# Supplementary figures and images for: Integrating CRISPR-Enabled Trackable Genome Engineering and Transcriptomic Analysis of Global Regulators for Antibiotic Resistance Selection and Identification in Escherichia coli
Source: mSystems. 2020 Apr 21;5(2):e00232-20. doi: 10.1128/mSystems.00232-20 (PMC7174635; doi:10.1128/mSystems.00232-20)

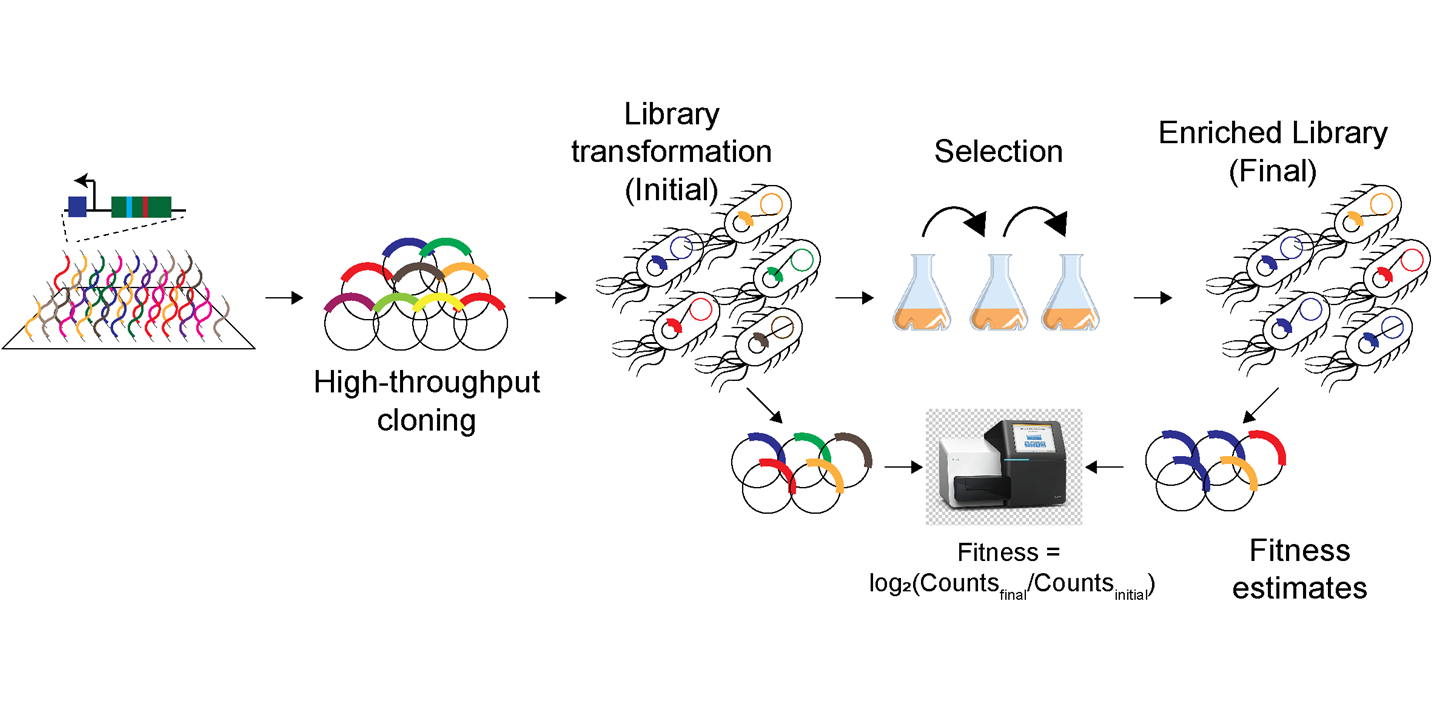

Supplement: FIG S1 [file mSystems.00232-20-sf001.tif]

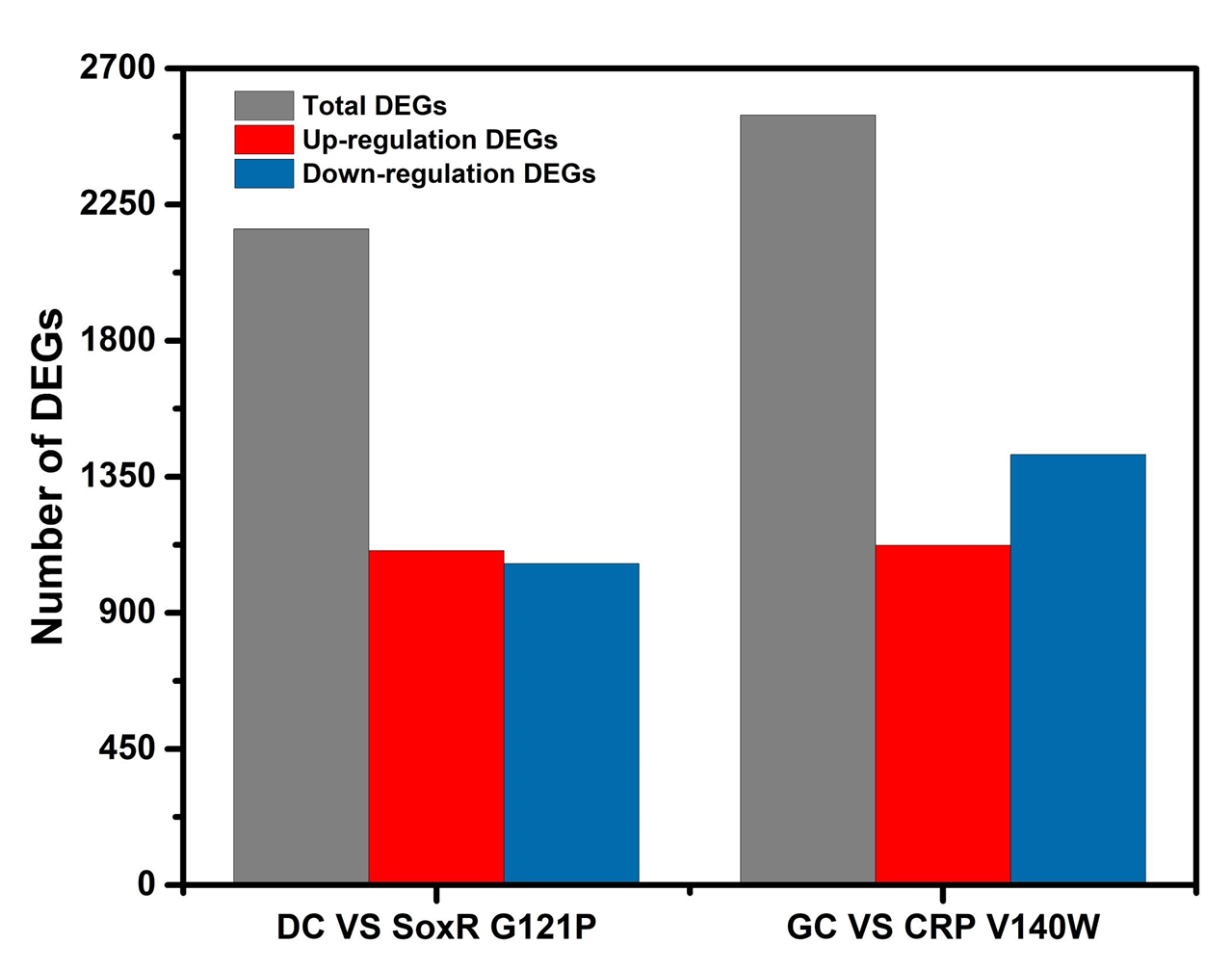

Supplement: FIG S2 [file mSystems.00232-20-sf002.tif]
