# Supplementary material for: Integrating CRISPR-Enabled Trackable Genome Engineering and Transcriptomic Analysis of Global Regulators for Antibiotic Resistance Selection and Identification in Escherichia coli
Source: mSystems. 2020 Apr 21;5(2):e00232-20. doi: 10.1128/mSystems.00232-20 (PMC7174635; doi:10.1128/mSystems.00232-20)
Supplement: TABLE S2 [file mSystems.00232-20-st002.docx]

**TABLE S2** The transformation efficiency of libraries.

| Library | Total number of colonies | Library size | Coverage | Transformation efficiency  (colonies/μg DNA) |
| --- | --- | --- | --- | --- |
| G1 | 203000 | 7000 | 29 | 6.7×10^4^ |
| G2 | 998240 | 7340 | 136 | 3.3×10^5^ |
| G3 | 259000 | 7400 | 35 | 8.6×10^4^ |
| G4 | 999400 | 5260 | 190 | 3.3×10^5^ |
| G5 | 293600 | 7340 | 40 | 9.7×10^4^ |
