# Supplementary material for: Integrating CRISPR-Enabled Trackable Genome Engineering and Transcriptomic Analysis of Global Regulators for Antibiotic Resistance Selection and Identification in Escherichia coli
Source: mSystems. 2020 Apr 21;5(2):e00232-20. doi: 10.1128/mSystems.00232-20 (PMC7174635; doi:10.1128/mSystems.00232-20)
Supplement: TABLE S3 [file mSystems.00232-20-st003.docx]

**TABLE S3** Summary of RNA-seq data.

|  | **Samples** | **Total reads**  **(raw data)** | **Total reads**  **(clean data)** | **Mapped reads** | **Detected genes** | **Detected genes/Total genes (%)** |
| --- | --- | --- | --- | --- | --- | --- |
| **Doxycycline** | DC-1 | 25405836 | 25297648 | 25205835 | 4409 | 98.02 |
|  | DC-2 | 19256942 | 19130364 | 19048234 | 4406 | 97.95 |
|  | DC-3 | 21105942 | 20969712 | 20893056 | 4401 | 97.84 |
|  | SoxR G121P-1 | 21156652 | 21031944 | 20938631 | 4341 | 96.51 |
|  | SoxR G121P-2 | 21803060 | 21659324 | 21571653 | 4344 | 96.58 |
|  | SoxR G121P-3 | 21349502 | 21218698 | 21128980 | 4346 | 96.62 |
| **Gentamicin** | GC-1 | 19368002 | 19291316 | 19207324 | 4426 | 98.40 |
|  | GC-2 | 20886556 | 20814206 | 20755148 | 4429 | 98.47 |
|  | GC-3 | 22727798 | 22647286 | 22570960 | 4438 | 98.67 |
|  | Crp V140W-1 | 20305428 | 20224684 | 16374998 | 4344 | 96.58 |
|  | Crp V140W-2 | 25013814 | 24907660 | 20893423 | 4355 | 96.82 |
|  | Crp V140W-3 | 21074270 | 20971406 | 17358110 | 4333 | 96.33 |
