# Supplementary material for: Integrating CRISPR-Enabled Trackable Genome Engineering and Transcriptomic Analysis of Global Regulators for Antibiotic Resistance Selection and Identification in Escherichia coli
Source: mSystems. 2020 Apr 21;5(2):e00232-20. doi: 10.1128/mSystems.00232-20 (PMC7174635; doi:10.1128/mSystems.00232-20)
Supplement: TABLE S4 [file mSystems.00232-20-st004.docx]

**TABLE S4** Relative expression levels of genes in ribosome synthesis, carbohydrate metabolism and oxidative phosphorylation affected by SoxR G121P responding to doxycycline.

| **Ribosome** | | | | |
| --- | --- | --- | --- | --- |
| **GeneName** | **Fold change** | **pvalue** | **padj** | **Name of the gene product** |
| *rpsR* | -25.93 | 1.52E-115 | 1.93E-114 | 30S ribosomal subunit protein S18 |
| *rpsF* | -18.45 | 0.00E+00 | 0.00E+00 | 30S ribosomal subunit protein S6 |
| *rpsS* | -18.10 | 6.00E-298 | 2.00E-296 | 30S ribosomal subunit protein S19 |
| *rpsJ* | -16.73 | 1.04E-187 | 2.23E-186 | 30S ribosomal subunit protein S10 |
| *rpsC* | -16.13 | 8.17E-225 | 2.19E-223 | 30S ribosomal subunit protein S3 |
| *rpsD* | -9.81 | 3.00E-151 | 5.22E-150 | 30S ribosomal subunit protein S4 |
| *rpsQ* | -8.16 | 2.91E-106 | 3.30E-105 | 30S ribosomal subunit protein S17 |
| *rpsK* | -7.38 | 3.90E-119 | 5.09E-118 | 30S ribosomal subunit protein S11 |
| *rpsG* | -7.31 | 4.42E-149 | 7.57E-148 | 30S ribosomal subunit protein S7 |
| *rpsB* | -6.59 | 7.48E-200 | 1.72E-198 | 30S ribosomal subunit protein S2 |
| *rpsL* | -6.36 | 5.53E-159 | 1.00E-157 | 30S ribosomal subunit protein S12 |
| *rpsE* | -5.50 | 2.52E-91 | 2.35E-90 | 30S ribosomal subunit protein S5 |
| *rpsI* | -5.36 | 1.30E-90 | 1.20E-89 | 30S ribosomal subunit protein S9 |
| *rpsM* | -5.29 | 2.99E-109 | 3.52E-108 | 30S ribosomal subunit protein S13 |
| *rpsH* | -5.07 | 3.88E-98 | 3.99E-97 | 30S ribosomal subunit protein S8 |
| *rpsN* | -4.54 | 1.35E-91 | 1.28E-90 | 30S ribosomal subunit protein S14 |
| *rpsP* | -3.74 | 3.81E-112 | 4.65E-111 | 30S ribosomal subunit protein S16 |
| *rpsA* | -3.46 | 2.96E-63 | 1.96E-62 | 30S ribosomal subunit protein S1 |
| *rplW* | -21.54 | 1.42E-235 | 4.06E-234 | 50S ribosomal subunit protein L23 |
| *rplB* | -20.53 | 2.47E-196 | 5.59E-195 | 50S ribosomal subunit protein L2 |
| *rplD* | -20.44 | 7.84E-205 | 1.86E-203 | 50S ribosomal subunit protein L4 |
| *rplV* | -16.94 | 1.14E-254 | 3.55E-253 | 50S ribosomal subunit protein L22 |
| *rplI* | -16.12 | 0.00E+00 | 0.00E+00 | 50S ribosomal subunit protein L9 |
| *rplP* | -12.27 | 4.20E-168 | 7.92E-167 | 50S ribosomal subunit protein L16 |
| *rplA* | -11.86 | 2.49E-261 | 8.25E-260 | 50S ribosomal subunit protein L1 |
| *rplK* | -11.79 | 0.00E+00 | 0.00E+00 | 50S ribosomal subunit protein L11 |
| *rpmC* | -10.90 | 8.60E-135 | 1.32E-133 | 50S ribosomal subunit protein L29 |
| *rplY* | -9.55 | 3.00E-302 | 1.00E-300 | 50S ribosomal subunit protein L25 |
| *rplQ* | -8.85 | 1.16E-117 | 1.50E-116 | 50S ribosomal subunit protein L17 |
| *rplL* | -8.16 | 6.23E-157 | 1.10E-155 | 50S ribosomal subunit protein L7/L12 |
| *rplJ* | -7.87 | 3.14E-210 | 7.70E-209 | 50S ribosomal subunit protein L10 |
| *rplO* | -5.86 | 1.21E-94 | 1.18E-93 | 50S ribosomal subunit protein L15 |
| *rplR* | -5.79 | 9.38E-109 | 1.10E-107 | 50S ribosomal subunit protein L18 |
| *rplF* | -5.56 | 1.05E-98 | 1.08E-97 | 50S ribosomal subunit protein L6 |
| *rpmH* | -4.86 | 4.48E-109 | 5.28E-108 | 50S ribosomal subunit protein L34 |
| *rplM* | -4.49 | 4.66E-95 | 4.59E-94 | 50S ribosomal subunit protein L13 |
| *rpmJ* | -4.07 | 9.72E-61 | 6.18E-60 | 50S ribosomal subunit protein L36 |
| *rpmA* | -4.02 | 4.13E-48 | 2.20E-47 | 50S ribosomal subunit protein L27 |
| *rplU* | -4.00 | 7.46E-136 | 1.16E-134 | 50S ribosomal subunit protein L21 |
| *rplE* | -3.90 | 2.16E-75 | 1.63E-74 | 50S ribosomal subunit protein L5 |
| *rpmD* | -3.86 | 5.70E-24 | 1.94E-23 | 50S ribosomal subunit protein L30 |
| *rplS* | -3.76 | 1.74E-92 | 1.65E-91 | 50S ribosomal subunit protein L19 |
| *rpmG* | -3.66 | 2.04E-36 | 8.86E-36 | 50S ribosomal subunit protein L33 |
| *rpmF* | -3.50 | 2.69E-37 | 1.18E-36 | 50S ribosomal subunit protein L32 |
| *rpmE* | -2.91 | 1.63E-53 | 9.48E-53 | 50S ribosomal subunit protein L31 |
| *rplX* | -2.79 | 5.13E-42 | 2.43E-41 | 50S ribosomal subunit protein L24 |
| *rplN* | -2.63 | 2.89E-45 | 1.48E-44 | 50S ribosomal subunit protein L14 |
| **Carbohydrate metabolism** | | | | |
| **GeneName** | **Fold change** | **pvalue** | **padj** | **Name of the gene product** |
| *fbaB* | 106.86 | 0.00E+00 | 0.00E+00 | fructose-bisphosphate aldolase class I |
| *pgi* | 10.24 | 3.61E-226 | 9.79E-225 | phosphohexose isomerase |
| *fbaA* | 8.49 | 1.95E-176 | 3.79E-175 | fructose-bisphosphate aldolase class II |
| *pfkB* | 5.65 | 9.10E-184 | 1.88E-182 | 6-phosphofructokinase II |
| *glpX* | 4.91 | 2.57E-135 | 3.97E-134 | fructose 6-bisphosphatase II |
| *pykA* | 4.68 | 1.25E-124 | 1.74E-123 | pyruvate kinase II |
| *eno* | 3.76 | 2.96E-95 | 2.93E-94 | enolase |
| *tpiA* | 3.74 | 5.74E-101 | 6.09E-100 | triosephosphate isomerase |
| *glk* | 3.68 | 9.94E-83 | 8.30E-82 | glucokinase |
| *pgk* | 3.54 | 4.54E-97 | 4.58E-96 | phosphoglycerate kinase |
| *gapA* | 3.10 | 3.41E-49 | 1.85E-48 | glyceraldehyde-3-phosphate dehydrogenase A |
| *pykF* | 3.00 | 4.67E-59 | 2.90E-58 | pyruvate kinase I |
| *ytjC* | 2.34 | 2.95E-24 | 1.01E-23 | phosphatase |
| *gpmA* | 2.32 | 1.25E-46 | 6.48E-46 | phosphoglyceromutase I |
| *pfkA* | -44.20 | 1.38E-221 | 3.62E-220 | 6-phosphofructokinase I |
| *aceE* | -3.82 | 2.73E-70 | 1.95E-69 | pyruvate dehydrogenase |
| *aceF* | -2.57 | 2.34E-43 | 1.14E-42 | pyruvate dehydrogenase |
| *lpd* | -2.22 | 4.45E-30 | 1.71E-29 | dihydrolipoyl dehydrogenase |
| *talA* | 80.72 | 0.00E+00 | 0.00E+00 | transaldolase A |
| *tktB* | 60.17 | 0.00E+00 | 0.00E+00 | transketolase B |
| *pgl* | 2.68 | 7.22E-38 | 3.21E-37 | 6-phosphogluconolactonase |
| *zwf* | 2.19 | 1.43E-28 | 5.32E-28 | glucose-6-phosphate 1-dehydrogenase |
| *rpe* | -2.34 | 3.84E-24 | 1.32E-23 | D-ribulose-5-phosphate 3-epimerase |
| *fumC* | 76.81 | 0.00E+00 | 0.00E+00 | fumarate hydratase |
| *sucD* | 17.50 | 4.44E-284 | 1.80E-282 | succinyl-CoA synthetase |
| *acnA* | 17.49 | 2.03E-217 | 5.20E-216 | aconitate hydratase |
| *sucA* | 14.18 | 1.26E-273 | 4.83E-272 | 2-oxoglutarate decarboxylase |
| *sucC* | 12.71 | 1.86E-128 | 3.04E-258 | spermidine/putrescine ABC transporter ATPase |
| *sucB* | 10.75 | 7.39E-230 | 3.04E-228 | dihydrolipoyltranssuccinase |
| *sdhD* | 7.16 | 9.73E-35 | 4.07E-34 | succinate dehydrogenase |
| *sdhC* | 6.06 | 6.14E-14 | 1.58E-13 | succinate dehydrogenase |
| *sdhA* | 5.68 | 6.53E-106 | 7.37E-105 | succinate dehydrogenase |
| *fumA* | 5.65 | 3.15E-103 | 3.44E-102 | fumarate hydratase |
| *sdhB* | 5.37 | 1.15E-77 | 8.98E-77 | succinate dehydrogenase |
| *frdC* | 4.64 | 1.05E-18 | 3.11E-18 | fumarate reductase |
| *frdD* | 4.13 | 1.73E-23 | 5.83E-23 | fumarate reductase |
| *frdA* | 4.12 | 8.93E-105 | 9.98E-104 | fumarate reductase |
| *frdB* | 3.18 | 6.12E-28 | 2.25E-27 | fumarate reductase |
| *gltA* | 3.09 | 6.85E-60 | 4.28E-59 | citrate synthase |
| *mdh* | 2.59 | 1.48E-46 | 7.69E-46 | malate dehydrogenase |
| *mqo* | -15.55 | 2.55E-193 | 5.72E-192 | malate dehydrogenase |
| *ybhJ* | -2.05 | 3.38E-06 | 6.34E-06 | aconitase family protein |
| *aceA* | 13.08 | 1.76E-205 | 4.23E-204 | isocitrate lyase |
| *aceB* | 12.00 | 5.28E-193 | 1.18E-191 | malate synthase A |
| *glcB* | 7.02 | 5.18E-103 | 5.63E-102 | malate synthase G |
| *acs* | 57.53 | 8.54E-281 | 3.37E-279 | acetyl-CoA synthetase |
| *ppc* | 2.26 | 8.04E-40 | 3.65E-39 | phosphoenolpyruvate carboxylase |
| *ackA* | 2.13 | 1.01E-27 | 3.69E-27 | acetate kinase |
| **Oxidative phosphorylation** | | | | |
| **GeneName** | **Fold change** | **pvalue** | **padj** | **Name of the gene product** |
| *sdhD* | 7.16 | 9.73E-35 | 4.07E-34 | succinate dehydrogenase |
| *sdhC* | 6.06 | 6.14E-14 | 1.58E-13 | succinate dehydrogenase |
| *sdhA* | 5.68 | 6.53E-106 | 7.37E-105 | succinate dehydrogenase |
| *sdhB* | 5.37 | 1.15E-77 | 8.98E-77 | succinate dehydrogenase |
| *cydA* | 4.75 | 8.77E-125 | 1.24E-123 | cytochrome d terminal oxidase subunit I |
| *frdC* | 4.64 | 1.05E-18 | 3.11E-18 | fumarate reductase |
| *frdD* | 4.13 | 1.73E-23 | 5.83E-23 | fumarate reductase |
| *frdA* | 4.12 | 8.93E-105 | 9.98E-104 | fumarate reductase |
| *ppk* | 4.08 | 1.44E-112 | 1.77E-111 | polyphosphate kinase |
| *nuoK* | 3.53 | 9.28E-07 | 1.79E-06 | NADH:ubiquinone oxidoreductase subunit K |
| *nuoN* | 3.51 | 1.49E-40 | 6.87E-40 | NADH:ubiquinone oxidoreductase subunit N |
| *nuoJ* | 3.39 | 7.76E-26 | 2.74E-25 | NADH:ubiquinone oxidoreductase subunit J |
| *nuoH* | 3.35 | 3.23E-47 | 1.70E-46 | NADH:ubiquinone oxidoreductase subunit H |
| *frdB* | 3.18 | 6.12E-28 | 2.25E-27 | fumarate reductase |
| *nuoI* | 3.11 | 2.24E-23 | 7.55E-23 | NADH-quinone oxidoreductase subunit I |
| *cydB* | 2.91 | 2.06E-37 | 9.07E-37 | cytochrome d terminal oxidase subunit II |
| *nuoL* | 2.91 | 6.37E-27 | 2.30E-26 | NADH:ubiquinone oxidoreductase subunit L |
| *nuoG* | 2.68 | 2.88E-50 | 1.60E-49 | NADH:ubiquinone oxidoreductase subunit G |
| *nuoM* | 2.58 | 1.16E-24 | 4.02E-24 | NADH:ubiquinone oxidoreductase subunit M |
| *nuoA* | 2.18 | 4.93E-20 | 1.51E-19 | NADH:ubiquinone oxidoreductase subunit A |
| *atpH* | -4.51 | 1.75E-62 | 1.14E-61 | F1 sector of membrane-bound ATP synthase delta subunit |
| *atpF* | -4.38 | 2.49E-70 | 1.78E-69 | F0 sector of membrane-bound ATP synthase subunit b |
| *ndh* | -3.66 | 7.15E-14 | 1.84E-13 | NADH dehydrogenase II |
| *atpG* | -3.62 | 1.59E-45 | 8.19E-45 | F1 sector of membrane-bound ATP synthase gamma subunit |
| *atpB* | -3.44 | 5.05E-54 | 2.96E-53 | F0 sector of membrane-bound ATP synthase subunit a |
| *atpA* | -3.34 | 8.84E-41 | 4.10E-40 | F1 sector of membrane-bound ATP synthase alpha subunit |
| *atpE* | -3.31 | 5.38E-33 | 2.17E-32 | F0 sector of membrane-bound ATP synthasesubunit c |
| *atpD* | -2.88 | 4.79E-35 | 2.01E-34 | F1 sector of membrane-bound ATP synthase beta subunit |
