# Supplementary material for: Integrating CRISPR-Enabled Trackable Genome Engineering and Transcriptomic Analysis of Global Regulators for Antibiotic Resistance Selection and Identification in Escherichia coli
Source: mSystems. 2020 Apr 21;5(2):e00232-20. doi: 10.1128/mSystems.00232-20 (PMC7174635; doi:10.1128/mSystems.00232-20)
Supplement: TABLE S5 [file mSystems.00232-20-st005.docx]

**TABLE S5** Relative expression levels of genes in biosynthesis of amino acids and fatty acid degradation affected by CRP V140W responding to gentamicin.

| **Amino acid synthesis** | | | | |
| --- | --- | --- | --- | --- |
| **GeneName** | **Fold change** | **pvalue** | **padj** | **Name of the gene product** |
| *leuC* | 48.45 | 2.52E-238 | 1.24E-236 | 3-isopropylmalate dehydratase large subunit |
| *leuB* | 41.74 | 0.00E+00 | 0.00E+00 | 3-isopropylmalate dehydrogenase |
| *leuA* | 40.36 | 1.98E-271 | 1.43E-269 | 2-isopropylmalate synthase |
| *tktB* | 36.99 | 4.36E-284 | 3.49E-282 | transketolase B |
| *talA* | 28.78 | 4.09E-221 | 1.59E-219 | transaldolase A |
| *leuD* | 25.36 | 5.74E-222 | 2.27E-220 | 3-isopropylmalate dehydratase small subunit |
| *lysA* | 15.73 | 9.23E-132 | 1.49E-130 | diaminopimelate decarboxylase |
| *pheA* | 12.95 | 0.00E+00 | 0.00E+00 | chorismate mutase and prephenate dehydratase |
| *pfkB* | 11.09 | 0.00E+00 | 0.00E+00 | 6-phosphofructokinase II |
| *thrA* | 10.99 | 2.92E-207 | 1.01E-205 | Bifunctional aspartokinase/homoserine dehydrogenase I |
| *glnA* | 9.91 | 1.05E-12 | 2.09E-12 | glutamine synthetase |
| *serC* | 8.28 | 2.02E-174 | 5.39E-173 | 3-phosphoserine/ phosphohydroxythreonine aminotransferase |
| *aroA* | 7.09 | 2.20E-77 | 1.77E-76 | 5-enolpyruvylshikimate-3-phosphate synthetase |
| *serA* | 6.96 | 1.38E-176 | 3.73E-175 | D-3-phosphoglycerate dehydrogenase |
| *cysK* | 6.75 | 4.31E-93 | 4.26E-92 | cysteine synthase A |
| *ilvN* | 6.75 | 1.80E-47 | 8.70E-47 | acetolactate synthase I small subunit |
| *aroG* | 6.71 | 1.17E-250 | 6.70E-249 | 3-deoxy-D-arabino-heptulosonate-7-phosphate synthase |
| *cysM* | 6.50 | 1.48E-175 | 3.96E-174 | cysteine synthase B |
| *aroE* | 5.92 | 2.09E-183 | 5.89E-182 | dehydroshikimate reductase |
| *thrB* | 5.66 | 1.82E-90 | 1.73E-89 | homoserine kinase |
| *luxS* | 5.51 | 2.01E-132 | 3.30E-131 | S-ribosylhomocysteine lyase |
| *asnA* | 5.44 | 4.92E-51 | 2.55E-50 | asparagine synthetase A |
| *argE* | 5.04 | 1.85E-117 | 2.54E-116 | acetylornithine deacetylase |
| *ltaE* | 4.84 | 7.81E-148 | 1.52E-146 | L-allo-threonine aldolase |
| *trpC* | 4.47 | 2.72E-68 | 1.90E-67 | indole-3-glycerol phosphate synthase / phosphoribosylanthranilate isomerase |
| *aroC* | 4.44 | 1.37E-90 | 1.30E-89 | chorismate synthase |
| *hisC* | 4.36 | 8.42E-54 | 4.60E-53 | histidinol-phosphate aminotransferase |
| *sdaA* | 4.31 | 2.02E-82 | 1.78E-81 | L-serine dehydratase I |
| *ilvB* | 4.01 | 2.75E-29 | 8.93E-29 | acetolactate synthase II large subunit |
| *gltB* | 3.92 | 8.88E-90 | 8.38E-89 | glutamate synthase |
| *hisF* | 3.87 | 6.27E-34 | 2.26E-33 | imidazole glycerol phosphate synthase |
| *aroH* | 3.85 | 1.80E-98 | 1.90E-97 | 3-deoxy-D-arabino-heptulosonate-7-phosphate synthase |
| *hisD* | 3.53 | 2.08E-66 | 1.42E-65 | bifunctional histidinal dehydrogenase/ histidinol dehydrogenase |
| *dapA* | 3.34 | 3.13E-100 | 3.46E-99 | dihydrodipicolinate synthase |
| *trpB* | 3.34 | 3.10E-41 | 1.29E-40 | tryptophan synthase |
| *hisB* | 3.31 | 3.07E-47 | 1.48E-46 | histidinol-phosphatase and imidazoleglycerol-phosphate dehydratase |
| *dapF* | 3.22 | 1.47E-65 | 9.82E-65 | diaminopimelate epimerase |
| *ilvM* | 3.18 | 1.94E-02 | 2.38E-02 | acetolactate synthase II small subunit |
| *thrC* | 3.17 | 2.12E-74 | 1.61E-73 | L-threonine synthase |
| *prs* | 3.08 | 1.65E-44 | 7.51E-44 | phosphoribosylpyrophosphate synthase |
| *hisA* | 3.08 | 9.59E-28 | 2.98E-27 | phosphoribosylformimino-5-aminoimidazole carboxamide ribotide isomerase |
| *proC* | 3.07 | 9.74E-61 | 5.91E-60 | pyrroline-5-carboxylate reductase |
| *hisH* | 3.06 | 8.91E-28 | 2.77E-27 | imidazole glycerol phosphate synthase |
| *dapD* | 2.98 | 3.42E-29 | 1.11E-28 | 2,3,4,5-tetrahydropyridine-2,6-dicarboxylate N-succinyltransferase |
| *trpA* | 2.91 | 1.42E-23 | 3.95E-23 | tryptophan synthase |
| *trpD* | 2.90 | 1.38E-11 | 2.67E-11 | Anthranilate synthase/ phosphoribosyl transferase |
| *ilvE* | 2.90 | 3.75E-33 | 1.33E-32 | branched-chain amino acid aminotransferase |
| *proA* | 2.84 | 4.21E-62 | 2.65E-61 | gamma-glutamylphosphate reductase |
| *aroK* | 2.79 | 1.56E-46 | 7.34E-46 | shikimate kinase I |
| *gltD* | 2.77 | 2.21E-20 | 5.64E-20 | glutamate synthase |
| *argH* | 2.74 | 2.41E-28 | 7.63E-28 | argininosuccinate lyase |
| *asd* | 2.74 | 1.53E-71 | 1.11E-70 | aspartate-semialdehyde dehydrogenase |
| *aroB* | 2.73 | 3.91E-73 | 2.92E-72 | 3-dehydroquinate synthase |
| *eno* | 2.72 | 1.62E-11 | 3.12E-11 | enolase |
| *ytjC* | 2.49 | 1.90E-47 | 9.17E-47 | phosphatase |
| *metC* | 2.47 | 9.04E-19 | 2.20E-18 | cystathionine beta-lyase |
| *aroD* | 2.44 | 1.26E-27 | 3.89E-27 | 3-dehydroquinate dehydratase |
| *acnA* | 2.42 | 1.31E-33 | 4.68E-33 | aconitate hydratase |
| *ilvD* | 2.30 | 3.79E-22 | 1.02E-21 | dihydroxyacid dehydratase |
| *alaA* | 2.27 | 2.79E-39 | 1.12E-38 | glutamate-pyruvate aminotransferase |
| *serB* | 2.27 | 5.55E-26 | 1.65E-25 | 3-phosphoserine phosphatase |
| *argG* | 2.26 | 6.17E-41 | 2.55E-40 | argininosuccinate synthetase |
| *pykA* | 2.25 | 6.30E-09 | 1.09E-08 | pyruvate kinase II |
| *dapB* | 2.22 | 4.10E-33 | 1.45E-32 | dihydrodipicolinate reductase |
| *rpe* | 2.16 | 1.63E-31 | 5.53E-31 | D-ribulose-5-phosphate 3-epimerase |
| *tdcB* | -448.77 | 5.01E-48 | 2.44E-47 | L-threonine dehydratase |
| *pfkA* | -89.48 | 1.49E-154 | 3.25E-153 | 6-phosphofructokinase I |
| *tdcG* | -14.75 | 7.53E-08 | 1.24E-07 | L-serine dehydratase |
| *yagE* | -8.94 | 2.81E-28 | 8.88E-28 | 2-dehydro-3-deoxy-D-pentonate aldolase |
| *ybhJ* | -7.30 | 1.34E-82 | 1.18E-81 | aconitase family protein |
| *metK* | -5.16 | 2.17E-183 | 6.08E-182 | S-adenosylmethionine synthetase |
| *metA* | -5.11 | 1.11E-45 | 5.14E-45 | homoserine O-succinyltransferase/ O-acetyltransferase |
| *argA* | -5.07 | 8.07E-98 | 8.49E-97 | amino-acid N-acetyltransferase |
| *aroF* | -4.78 | 5.52E-136 | 9.37E-135 | 3-deoxy-D-arabino-heptulosonate-7-phosphate synthase |
| *yjhH* | -4.35 | 1.71E-16 | 3.87E-16 | putative lyase/synthase |
| *argI* | -4.28 | 1.95E-10 | 3.62E-10 | ornithine carbamoyltransferase I |
| *gltA* | -3.85 | 2.04E-42 | 8.72E-42 | citrate synthase |
| *tyrA* | -3.80 | 5.90E-117 | 7.98E-116 | fused chorismate mutase T/prephenate dehydrogenase |
| *metE* | -3.43 | 7.48E-42 | 3.17E-41 | 5-methyltetrahydropteroyltriglutamate-homocysteine S-methyltransferase |
| *argF* | -2.65 | 5.57E-22 | 1.49E-21 | ornithine carbamoyltransferase |
| *sdaB* | -2.62 | 1.46E-37 | 5.66E-37 | L-serine dehydratase II |
| *metB* | -2.32 | 1.94E-28 | 6.17E-28 | cystathionine gamma-synthase |
| *argC* | -2.24 | 6.51E-08 | 1.07E-07 | N-acetyl-gamma-glutamylphosphate reductase |
| **Fatty acid degradation** | | | | |
| **GeneName** | **Fold change** | **pvalue** | **padj** | **Name of the gene product** |
| *adhP* | 14.05 | 0.00E+00 | 0.00E+00 | ethanol-active dehydrogenase/acetaldehyde-active reductase |
| *adhE* | 4.96 | 1.71E-80 | 1.47E-79 | acetaldehyde dehydrogenase / alcohol dehydrogenase |
| *aas* | 4.16 | 4.82E-77 | 3.86E-76 | acyl-[acyl-carrier-protein]-phospholipid O-acyltransferase / long-chain-fatty-acid--[acyl-carrier-protein] ligase |
| *hcaD* | 2.04 | 2.17E-07 | 3.49E-07 | phenylpropionate dioxygenase |
| *yiaY* | -23.98 | 8.80E-10 | 1.59E-09 | 3-ketoacyl-CoA thiolase (thiolase I) |
| *fadB* | -21.58 | 1.85E-16 | 4.19E-16 | 3-hydroxyacyl-CoA dehydrogenase/ enoyl-CoA hydratase/ 3-hydroxybutyryl-CoA epimerase/ enoyl-CoA isomerase |
| *frmA* | -17.42 | 3.97E-21 | 1.04E-20 | L-threonine dehydrogenase |
| *fadE* | -9.85 | 4.65E-23 | 1.28E-22 | acyl-CoA dehydrogenase |
| *yqeF* | -6.72 | 2.00E-28 | 6.36E-28 | beta-ketoacyl-CoA thiolase |
| *fadI* | -4.22 | 4.68E-92 | 4.54E-91 | acyl-CoA synthetase (long-chain-fatty-acid-CoA ligase) |
| *fadD* | -3.98 | 2.27E-95 | 2.29E-94 | short chain acyltransferase |
| *fadA* | -2.64 | 2.10E-153 | 4.49E-152 | acyl coenzyme A dehydrogenase |
| *fadJ* | -2.39 | 0.00E+00 | 0.00E+00 | alcohol dehydrogenase class III |
