# Supplementary material for: Integrating CRISPR-Enabled Trackable Genome Engineering and Transcriptomic Analysis of Global Regulators for Antibiotic Resistance Selection and Identification in Escherichia coli
Source: mSystems. 2020 Apr 21;5(2):e00232-20. doi: 10.1128/mSystems.00232-20 (PMC7174635; doi:10.1128/mSystems.00232-20)
Supplement: TABLE S6 [file mSystems.00232-20-st006.docx]

**TABLE S6** Primers used in reconstruction of mutant strains.

| **Primers** | **Squence (5’ → 3’)** |
| --- | --- |
| pGRS-F | cacaccaggtctcACTGGACGGATGTATgttttagagctagaaatagcaagttaa |
| pGRS-R | cacaccaggtctcAccagttcgtcaactagtattatacctaggactgagc |
| pGRC-F | cacaccaggtctcAGCGTTCCTCGACGTGAgttttagagctagaaatagcaagttaa |
| pGRC-R | cacaccaggtctcAACGCCAGactagtattatacctaggactgagc |
| 121K-F1 | TCATCTGTTGGGGAGTATAA |
| 121K-R1 | GGCAGCCACATTTGATGCATCCGTCCAGTTCGTCACGCAGCG |
| 121K-F2 | GGATGCATCAAATGTGGCTGCCTTTCGCGCAGTGATTGCCCG |
| 121K-R2 | TGATGGCGTAGACATAAGAA |
| 120E-F1 | TCATCTGTTGGGGAGTATAA |
| 120E-R1 | GGCAGCCACAACCTTCGCATCCGTCCAGTTCGTCACGCAGCG |
| 120E-F2 | GGATGCGAAGGTTGTGGCTGCCTTTCGCGCAGTGATTGCCCG |
| 120E-R2 | TGATGGCGTAGACATAAGAA |
| 121N-F1 | TCATCTGTTGGGGAGTATAA |
| 121N-R1 | GGCAGCCACAGTTGATGCATCCGTCCAGTTCGTCACGCAGCG |
| 121N-F2 | GGATGCATCAACTGTGGCTGCCTTTCGCGCAGTGATTGCCCG |
| 121N-R2 | TGATGGCGTAGACATAAGAA |
| 121P-F1 | TCATCTGTTGGGGAGTATAA |
| 121P-R1 | GGCAGCCACACGGGATGCATCCGTCCAGTTCGTCACGCAGCG |
| 121P-F2 | GGATGCATCCCGTGTGGCTGCCTTTCGCGCAGTGATTGCCCG |
| 121P-R2 | TGATGGCGTAGACATAAGAA |
| 121I-F1 | TCATCTGTTGGGGAGTATAA |
| 121I-R1 | GGCAGCCACAAATGATGCATCCGTCCAGTTCGTCACGCAGCG |
| 121I-F2 | GGATGCATCATTTGTGGCTGCCTTTCGCGCAGTGATTGCCCG |
| 121I-R2 | TGATGGCGTAGACATAAGAA |
| 120D-F1 | TCATCTGTTGGGGAGTATAA |
| 120D-R1 | GGCAGCCACAACCATCGCATCCGTCCAGTTCGTCACGCAGCG |
| 120D-F2 | GGATGCGATGGTTGTGGCTGCCTTTCGCGCAGTGATTGCCCG |
| 120D-R2 | TGATGGCGTAGACATAAGAA |
| 140W-F1 | GGTGCTTTATCGCCTGAGTT |
| 140W-R1 | GTGCAATGCGGCCGGTCCAATCGAGGAACGCCAGGTTGCCCACTTTCTC |
| 140W-F2 | CTCGATTGGACCGGCCGCATTGCACAGACTCTGCTGAATCTGGCAAAACA |
| 140W-R2 | CATGTATCCCGCCAAACTGA |

^a^Single underline stands for the restriction site, *Bsa*I.

^b^Double underline stands for a sequence complementary to pGRB.
