# Supplementary material for: Integrating CRISPR-Enabled Trackable Genome Engineering and Transcriptomic Analysis of Global Regulators for Antibiotic Resistance Selection and Identification in Escherichia coli
Source: mSystems. 2020 Apr 21;5(2):e00232-20. doi: 10.1128/mSystems.00232-20 (PMC7174635; doi:10.1128/mSystems.00232-20)
Supplement: TABLE S7 [file mSystems.00232-20-st007.docx]

**TABLE S7** Primers used in qRT-PCR.

| **Primers** | **Squence (5’ → 3’)** |
| --- | --- |
| *soxS*-F | CGAGCATATTGACCAGCCGCTTAACAT |
| *soxS*-R | CTGCTGCGAGACATAACCCAGGT |
| *marR*-F | CTGGATATTACCGCGGCACAGTT |
| *marR*-R | CACATATTGCCGCGCCGCCGGT |
| *marB*-F | CATGAAACCACTTTCATCCGCAAT |
| *marB*-R | TAGCGTGTTGATTATAATAGGGCAC |
| *acrZ*-F | CTGTGCTTAGCGGTTAGAATAGT |
| *acrZ*-R | CGACTAAGCGGGCATTCAGGGA |
| *acrA*-F | CACCTCTCCGATTAGCGGTCGCAT |
| *acrA*-R | CGGGAACTTAATGCCGTCACTGGT |
| *acrB*-F | GCTTCTTCGGCTGGTTTAACCGCAT |
| *acrB*-R | CTGTGTACGTTCCTGCGTTGCACCT |
| *ompN*-F | CAATGGCGTCGCGACTTATCGTAAT |
| *ompN*-R | CATTGGTGCGGTCAGAAGAGGTGTAT |
| *ompF*-F | CGATGACTTCTTCGTTGGTCGTGT |
| *ompF*-R | CAGGTTGGTACGGTCAGCTGCACCAT |
| *sodA*-F | CTGATCACCAAACTGGACCAGCT |
| *sodA*-R | CACCAGCCATGCCCAGCCGGAAC |
| *fumC*-F | CACGTAGCGGAACTGGCTCTT |
| *fumC*-R | TGAACCAGGGCATCACAGGT |
| *rpsR*-F | GACTATAAAGATATCGCTACGCT |
| *rpsR*-R | GACAGGTAGCGAGCGCGTTTGAT |
| *rplD*-F | TGGCGTGACCTTTGCTGCTCGT |
| *rplD*-R | CAGTTTCTGTGCCAGCAGCTTAGT |
| *rpsF*-F | GCTGGCTTACCCGATCAACAAACT |
| *rpsF*-R | GCGTGCTTGGTACGCATAACCAT |
| *rpsC*-F | CGTGTAACCATTCACACTGCTCGC |
| *rpsC*-R | GAAGTGATGCTGTCAGCAACCAGT |
| *rpsQ*-F | GTGAAACACCCGATCTACGGT |
| *rpsQ*-R | GTCTTGGACAGCGGACGGCAT |
| *rpsB*-F | TCGTCAGTCCATCAAACGTCTG |
| *rpsB*-R | GCATCGATTACAAACAGAGCGT |
| *rplE*-F | GTCAGGGCTATCCGATCGGCT |
| *rplE*-R | CATGCTGTAGTTACCACGACCGT |
| *fbaB*-F | GCGTGGAGCAGGCGTTCAACAT |
| *fbaB*-R | CTTAAAGGCGGAGTTACGCAAAT |
| *pgi*-F | GTGGTCAGCGATTGGCCTGTCGAT |
| *pgi*-R | CAGATGCCAATCAGCGCCAGCAGT |
| *pfkA*-F | CTTCACTGCGCTGAGCACCGT |
| *pfkA*-R | CGAATTCACAGCCACCGGCAAT |
| *aceE*-F | GTGCCGGTGTCTGATGCAGATAT |
| *aceE*-R | CAGCTCAAGCTTCTCGGTGAAGT |
| *talA*-F | GAATTCGCGCGGCAGAAGAGCT |
| *talA*-R | GTGCCTGATACCAGTCATAAAT |
| *tktB*-F | GCAGAAGAGTTTACCCGACGGAT |
| *tktB*-R | GCCGAGCAACTCAGGCAGCAT |
| *sucD*-F | GAACTGCCCAGGCGTTATCACT |
| *sucD*-R | GACCGAAACCGTAATCCGTGGTCT |
| *acnA*-F | GTGGTCATTGCTGCGATAACCT |
| *acnA*-R | GCCAGATAATCAGAAACGACT |
| *sucA*-F | GTCTTCATCGACCTGGTGTGCT |
| *sucA*-R | GTTAACCATCTCGGTGGCATCT |
| *mqo*-F | GATTCCGGAAGCGAAAGACT |
| *mqo*-R | GTCCAGAACGCGGGTATCGATATGC |
| *aceA*-F | GTGCCAACTCAGGAAGCTATTC |
| *aceA*-R | CGCTGTCATACGGGTCGCAAT |
| *aceB*-F | CGTGTTCCAGATGGATGAAATCCT |
| *aceB*-R | GTGAGTAAGCATTCAGGAATGGT |
| *ndh*-F | CTGACCAACGAAGCCCTGAACGT |
| *ndh*-R | GCCATCTTTAGTGTGCAGGC |
| *gadB*-F | GAAGCCTGTGACGAAAACACCAT |
| *gadB*-R | GTGCATGTCGATGTCGATACC |
| *gadA*-F | GATGGCGATGAAATGGCGTT |
| *gadA*-R | CATAGGGATCTCACGCAGCTCCAC |
| *flgB*-F | GTCATGCAACGTGGACGGGAT |
| *flgB*-R | CGTTCGCGATCCATATCGACGGT |
| *fliA*-F | GCAACTGGAGCAGGAACTTGG |
| *fliA*-R | CCAGTTCGATGCTATCGCCGTGCT |
| *motA*-F | CGTCGATTATCTGCGCCTGATTAT |
| *motA*-R | CCATTACAGCCGCAACAATACC |
| *motB*-F | CAAAATCGATCTGGTCCAGGAAGGT |
| *motB*-R | GTATGACCTGAAAGGCTAATACGGT |
| *flhD*-F | GCGTCCGCTATGTTTCGTCT |
| *flhD*-R | CTGAGTAATCGTCTGGTGGCTGT |
| *flhC*-F | CATTCTCAACCGACTGGTTTATGAC |
| *flhC*-R | GGTTAATGCCAGCAGTGGTCCTT |
| *tar*-F | GACCTGGCGCAGAGCGTTTCACAT |
| *tar*-R | GGCAGTTTCTTCCAGCGCGGAT |
| *cheA*-F | GCAGGAATCAGTGATGTCGAT |
| *cheA*-R | GATAATGCGTTCTATCAGGCT |
| *pdeH*-F | GCTGGCATCGGTCAATATTGAT |
| *pdeH*-R | CAAATTCACACATCGAGGCAAAG |
| *flu*-F | CTCACTGTCAGCAACACCACACT |
| *flu*-R | CAATGGCACCGTTCAGCACAGT |
| *leuB*-F | GATACCGAGGTGTATCACCGTT |
| *leuB*-R | CAGTTCGACATCCGGGTATTCCGT |
| *leuA*-F | CTGTTCCCTGGAAGAAGTCATCAT |
| *leuA*-R | GTATACCGGAGGAGTGTGCGAAT |
| *serC*-F | CAGACTTCGGCGCAGATGTGGT |
| *serC*-R | CACGAACGATGACGATTGTCAG |
| *tdcB*-F | GTGAGCGAAATTGTCGAAATGGAAG |
| *tdcB*-R | CACGAATGGTCGGGTTAATAGAT |
| *fadB*-F | GACAAGTCGTTAACCCTCGGCAT |
| *fadB*-R | CAACAACCGCTTCTACCACAATAT |
| *yqeF*-F | CATCAGCCGTCAGTTGCAGGAT |
| *yqeF*-R | CTAAGCCTTCTGCGCTGGCGTCAGT |
| *sdhC*-F | GCATCCTGCTGTGGCTTCTGGGT |
| *sdhC*-R | CCATCATCATGTGGCGAATACCT |
